# Ion-Electron Coupling-Driven Redox Behavior in Metal–Organic Frameworks

**DOI:** 10.1021/jacs.6c03704

**Published:** 2026-04-21

**Authors:** A. Avilés, M. Ghotbi, A. J. Ferguson, J. L. Blackburn, A. A. Talin, M. Y. Darensbourg, P. B. Balbuena

**Affiliations:** † Department of Chemical Engineering, Texas A&M University, College Station, Texas 77843, United States; ‡ Department of Chemistry, Texas A&M University, College Station, Texas 77843 United States; § Department of Materials Science and Engineering, Texas A&M University, College Station, Texas 77843, United States; ∥ Chemistry and Nanoscience Center, National Laboratory of the Rockies (NLR), Golden, Colorado 80401, United States; ⊥ Chemistry, Combustion and Materials Science Department, Sandia National Laboratories, Livermore, California 94550, United States

## Abstract

Redox-active metal–organic frameworks (MOFs) have long been proposed as electronic transport platforms, yet the microscopic origin of their conductivity remains debated. A theoretical demonstration reveals charge transport in a Zn­(pyrazole–naphthalene diimide (NDI)) MOF arising not from delocalized band-like states but from redox hopping between discrete linker sites. Using ab initio molecular dynamics simulations combined with electronic structure analysis, we established a direct link among electron injection, structural reorganization, and transport. Electron accumulation proceeds sequentially and site-selectively from imide and carbonyl groups of the NDI core progressively involving pyrazole N atoms at higher reduction states, through a hierarchy of redox-active sites. In contrast, Zn nodes remain essentially redox-inactive, which confirms their structural role. Density-of-states analysis corroborates a transport regime dominated by linker-centered states with evolving p-character upon reduction, resulting in dynamically reconfigured conduction networks. Real-time trajectories reveal anisotropic linker-to-linker electron transfer modulated by counterion coordination. This cooperative ion–electron regime emerges from potential energy surface collapse into a single low-barrier transition (Δ*G* ^‡^ ≈ 45 meV), where ionic and electronic motions evolve adiabatically on the same free-energy landscape. Elucidating redox conductivity in Zn­(pyrazole–NDI) MOFs provides a theoretical framework for use in neuromorphic computing and related technologies.

## Graphical abstract

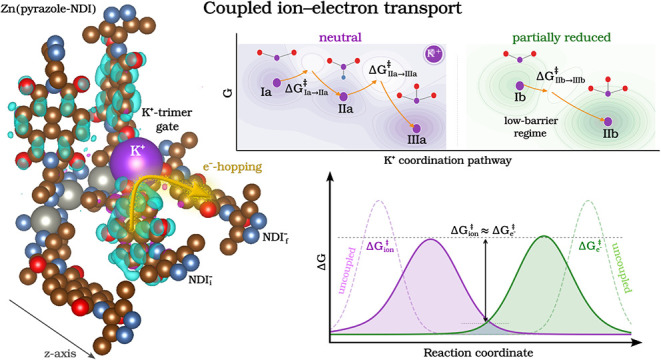

## Introduction

Metal–organic frameworks (MOFs) are a class of coordination polymers with permanent porosity, constructed via the assembly of organic linkers and metal nodes.^1,2^ Understanding the fundamental mechanisms of electronic transport in redox-active MOFs is critical for their use as functional electronic and optoelectronic materials, particularly for systems that can emulate synaptic and neuronal functions in neuromorphic computing and reconfigurable electronic devices.^3–6^ In MOFs, the interplay among framework topology, linker chemistry, and redox dynamics is expected to govern charge mobility, spatial delocalization, and response to external stimuli. Among various redox-active organic linkers, *naphthalene diimide* (NDI) is a planar aromatic building block with high thermal stability, chemical robustness, and significant electron (e^–^) affinity, enabling reversible redox activity.^7^ Electrochemical studies reveal two single-e^–^ reduction processes at −0.76 and −1.00 V (vs Ag/AgCl),^8^ yielding stable radical anions and dianions, respectively. These features make NDI a key building block for organic n-type semiconductors,^9,10^ field-effect transistors,^11,12^ and optoelectronic devices.^13–15^ Supramolecular assembly of NDI π-systems relies on noncovalent interactions,^16–18^ including π-stacking, hydrogen bonding (H-bonding), and van der Waals forces.^19^ These interactions enable the construction of organized architectures at multiple length scales, from the nano- to the macroscopic, using chromophoric building blocks. The controlled spatial arrangement of naphthalene imide units, and their fundamental capacity for efficient, photoinduced electron transfer (ET),^18^ allows for the optimization of photophysical^20,21^ and charge transport properties.^22^

Early examples of materials featuring π-conjugated imide moieties capable of self-assembly through supramolecular interactions yielded nanostructured semiconductor architectures that support unidirectional long-range charge transport.^23–25^ These assemblies offer properties such as nanoscale dimension control, stimuli responsiveness, and versatility in electronic behavior.^26^ For example, Li et al.^27^ developed oligothiophene–perylenediimide (OT–PDI) dyads forming well-defined donor–acceptor fibrous nanoassemblies by tuning hydrophobic and hydrophilic tails. These resulting bicontinuous nanostructured OT–PDI arrays demonstrate efficient e^–^ and hole transport. Transient absorption spectroscopy^28,29^ and flash-photolysis time-resolved microwave conductivity^30^ demonstrated that precisely designed OT–PDI assemblies exhibit higher conductivity and carrier generation efficiency than less ordered systems.

These results emphasize the importance of supramolecular design in optimizing the e^–^ transport. However, these architectures exhibit inherent limitations, such as thermal sensitivity,^31^ which can compromise operational performance and stability. Efficient electronic transport in these systems relies on precise control of supramolecular interactions, increasing design and fabrication complexity. High synthesis and assembly costs further limit the scalability for commercial applications. Additionally, the relationship between supramolecular structure and charge carrier transport remains unclear,^32,33^ and the underlying e^–^ transfer mechanisms are still unexplored. To address these challenges, Ott et al.^34^ proposed an alternative approach based on constructing electroactive MOFs from NDI derivatives.^35^ In this strategy, pyrazole–NDI linkers coordinate tetrahedral Zn^2+^ centers to form Zn­(pyrazole–NDI) (pyrazole–NDI = 1,4-bis­[(3,5-dimethyl)-pyrazole-4-yl]­naphthalenediimide), a framework designed to exploit the electroactivity and biocompatibility of NDI for applications in neuromorphic computing and reconfigurable electronics^36^ (Figure 1).

**Figure 1:**
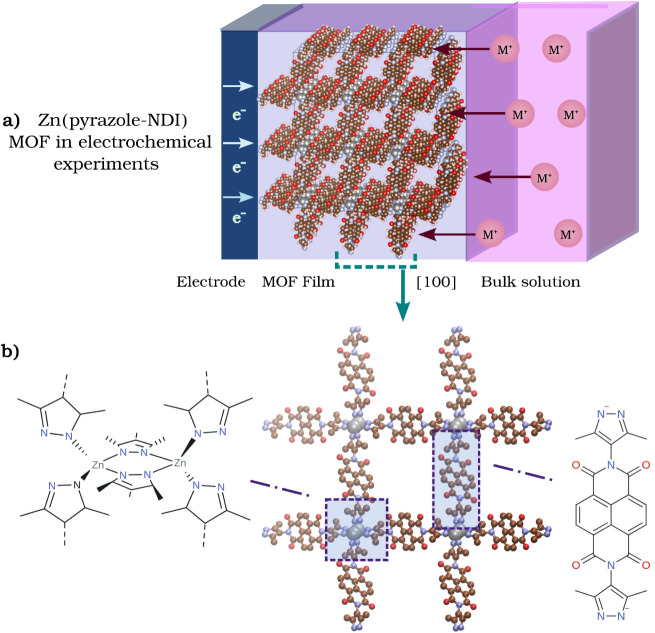
(a) Schematic of a Zn­(pyrazole–NDI) MOF film under electrochemical conditions, illustrating e^–^ injection from the electrode and the simultaneous migration of charge-compensating countercations (M^+^) from the bulk solution into the framework. (b) Crystal structure of Zn­(pyrazole–NDI) viewed along the [100] direction, highlighting Zn^2+^ nodes (gray spheres) coordinated by pyrazole–NDI linkers. This framework provides a model platform to study ion-coupled redox hopping and to derive key insights into charge transport for the design of neuromorphic devices.

MOFs are valued for their tunable structures, permanent porosity, and diverse electronic functionalities.^37^ They have been applied in gas separation and storage,^38,39^ catalysis,^40,41^ energy storage,^42^ and reconfigurable electronics, with Zn­(pyrazole–NDI) as a notable example.^34,43^ Several of these applications depend on efficient e^–^ transport. Conductivity in MOFs can arise through either band-like^44^ or hopping^45^ transport mechanisms. Band-like transport includes through-bond,^46^ extended conjugation,^47^ and through-space pathways,^48^ all requiring careful alignment of node–linker energy levels and optimized stacking geometry.^31,32,49^

Although redox-hopping MOFs generally exhibit lower conductivity (typically around 10^–7^ S·cm^–1^) than band-like transport MOFs (∼10^3^ S·cm^–1^),^50^ their transport response is not defined by a single fixed value but depends strongly on the framework’s redox state. In Zn­(pyrazole–NDI),^34^ for example, the conductivity spans up to 4 orders of magnitude and reaches its maximum under mixed-valence conditions. This behavior makes these materials especially attractive for electrocatalysis and reconfigurable electronics, including switching devices^51,52^ and nonvolatile memory.^53–55^

Its versatility arises from the ease of integrating redox hopping into MOF structures using simple design and experimental strategies.^56^ MOFs also allow precise structural modification,^57^ and postsynthetic methods enable facile functionalization^58^ or anchoring of redox centers,^43^ resulting in materials with diverse and multifunctional properties. Ott et al.^34^ prepared thin films (∼700 nm) of Zn­(pyrazole–NDI) on fluorine-doped tin oxide electrodes and performed slow-scan cyclic voltammetry, observing two well-defined, reversible reduction waves at −0.79 and −1.17 V (vs Ag/AgNO_3_), consistent with the formal potentials of the pyrazole–NDI linker. These redox waves correspond to sequential single-e^–^ reduction steps. The authors found that redox conductivity reaches its maximum when half of the NDI linkers are reduced to NDI•^–^ or when half of NDI•^–^ are further reduced to NDI•^2–^, reflecting the bimolecular nature of ET between adjacent linkers—requiring both reduced and oxidized sites (mixed valence conditions) for conduction. Moreover, it was suggested that conductivity in Zn­(pyrazole–NDI) shares microscopic characteristics with previously reported MOFs, such as charge carriers being localized at discrete redox sites^34,59,60^ and moving via thermally activated jumps closely associated with the migration of charge-balancing countercations.^61–64^ Ionic diffusion is the rate-limiting step in redox hopping, yet the detailed electrical transport mechanism in MOFs remains poorly understood. Nevertheless, critical gaps persist in elucidating how counterions modulate the evolving redox landscape, how thermal fluctuations perturb the Marcus hopping barriers, and how these microscopic dynamics integrate to produce the macroscopic transport response.

To address these gaps, we employ first-principles computational methods, density functional theory, and ab initio molecular dynamics simulations (DFT and AIMD), to reveal the atomistic basis of redox hopping in Zn­(pyrazole–NDI). We show that the Zn­(pyrazole–NDI) MOF displays a coordination environment that minimizes band dispersion, reduces d−π overlap, and limits π–π coupling between linkers, making redox hopping the dominant charge transport mechanism. Our simulations reveal that electron transport occurs through linker-to-linker hopping, in which an added e^–^ dynamically redistributes to preferential sites depending on the system’s level of reduction. This process induces bond-length modulations, dihedral angle fluctuations, and transient noncovalent interactions that, in turn, modulate the electronic accessibility and coupling of neighboring linkers, thereby impacting conductivity. AIMD supercell lattice trajectories show that the excess e^–^ does not remain localized but spontaneously migrates across multiple NDI linkers coordinated to the same Zn^2+^ node. This e^–^ migration proceeds along preferential anisotropic pathways dictated by the Zn–linker coordination geometry, with Zn nodes mediating interlinker electronic coupling despite remaining redox-inactive. Our simulations also show that potassium counterions actively participate in charge transport. By coordinating to NDI-carbonyl oxygens, K^+^ ions stabilize symmetry-broken charge distributions at half reduction [Zn­(pyrazole–NDI•^–0.5^)] and promote the formation of trimeric assemblies that reinforce inter-NDI connectivity, thereby enabling multidirectional transport. This interplay of site-specific redox activity, structural adaptability, and ion-mediated connectivity parallels the synaptic behavior in neuromorphic systems. Our findings establish new principles for engineering MOFs as reconfigurable materials for neuromorphic applications.

## Computational Details

DFT and AIMD simulations were performed with VASP using PAW potentials and the PBE functional to model the structural and redox responses of the Zn­(pyrazole–NDI) framework. AIMD trajectories were analyzed using PMF-based descriptors to quantify K^+^ migration barriers, and constrained DFT (CDFT) calculations in NWChem were employed to evaluate the Marcus parameters governing electron hopping. Full computational details, including basis sets, functional selections, and postprocessing workflows, are provided in the Supporting Information.

## Results and Discussion

### Electronic and Structural Architecture of the Zn­(pyrazole–NDI) MOF

The optimized crystal structure of Zn­(pyrazole–NDI), shown in Figure 1a and b, provides key insights into the spatial arrangement and connectivity that govern its electronic properties. The unit cell features NDI···NDI center-to-center distances of 9.05 Å, with interplanar separations along the *x*, *y*, and *z* axes of 6.19, 16.69, and 17.01 Å, respectively. The shortest separation of 6.19 Å lies well beyond the range typically associated with π–π stacking, indicating no significant direct orbital overlap between adjacent linkers. Here, “adjacent” refers to ligands whose extended π-systems are aligned along parallel unit cell edges and exhibit the shortest center-to-center distances within the crystal lattice. The tetrahedral geometry of Zn^2+^ nodes, highlighted in Figure 2a, arises from direct coordination to the ligands through pyrazole groups. This arrangement is typical of MOFs featuring tetrahedrally coordinated Zn^2+^ nodes that propagate into infinite one-dimensional chains.^65^ The average Zn–N bond length is 1.98 Å, and the Zn–Zn node separation is 3.20 Å.

**Figure 2:**
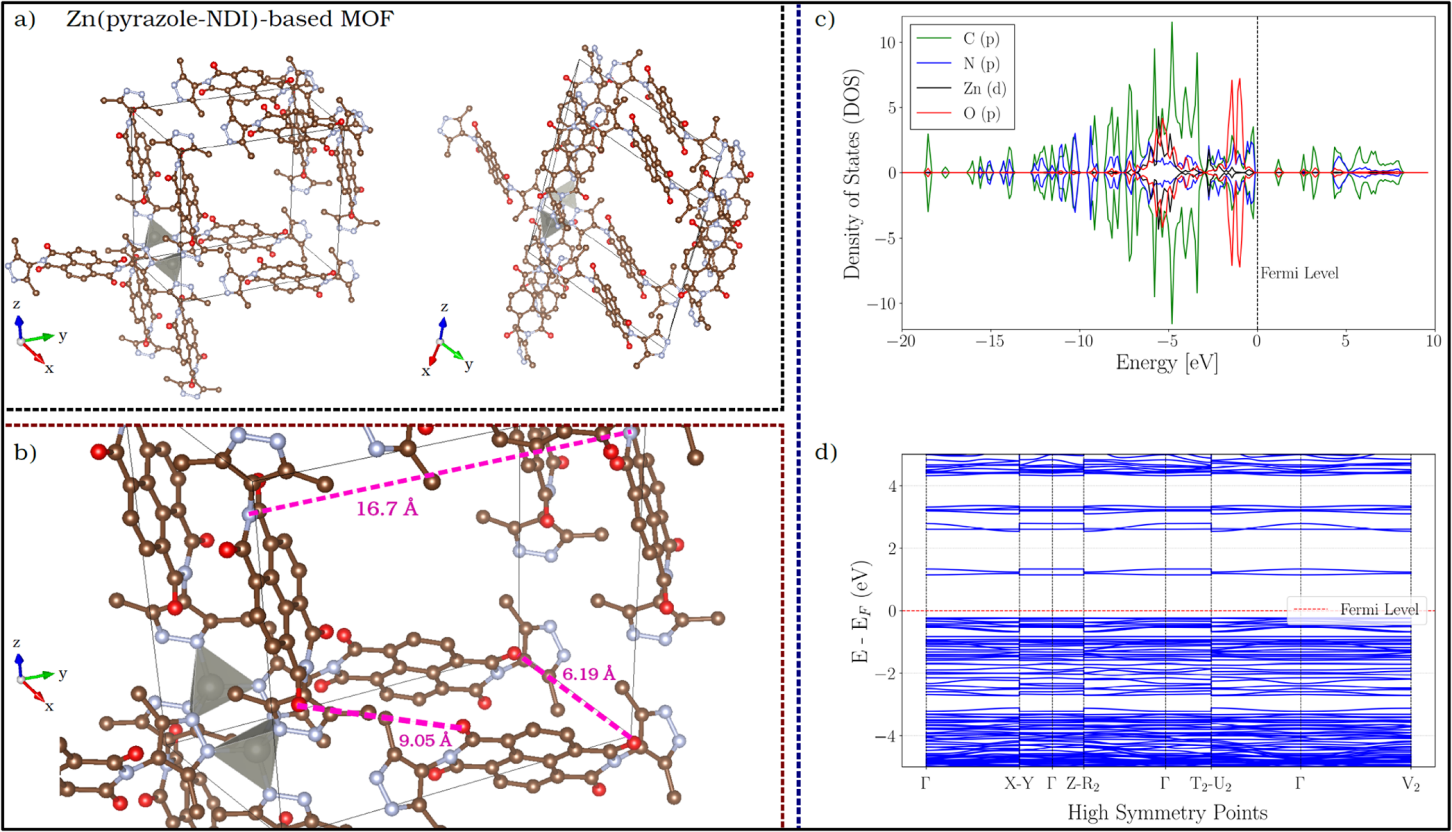
(a) Optimized unit cell of Zn­(pyrazole–NDI), illustrating the overall framework architecture from two complementary orthogonal perspectives. Pyrazole–NDI linkers and tetrahedral Zn^2+^ coordination nodes are shown. (b) Detailed close-up of the same unit cell, highlighting the optimized geometric parameters (closest interplanar distance: 6.19 Å—above the π–π stacking range). Colored spheres indicate atom types as follows: brown (C), blue (N), red (O), and gray (Zn); hydrogen atoms (H) have been omitted for clarity. (c) PDOS for the valence orbitals of C, N, O, and Zn. Energies are referenced to *E*_*f*_ = 0 eV; negative values correspond to occupied (valence) states and positive values to unoccupied (conduction) states. (d) Electronic band structure referenced to the Fermi level.

The optimized crystal lattice reveals that the pyrazole–NDI ligands exhibit aromatic π-systems with planar rings, characteristic of naphthalene diimide. In contrast, the pyrazole groups are displaced from this plane, forming dihedral angles that range from 79.6° to 92.4°. The π-systems of adjacent ligands are parallel in all spatial directions, although they do not share the same plane. This geometric configuration prevents direct intermolecular π-orbital overlap, confirming that redox hopping should be the primary mechanism of charge transport in this system.

Projected and total density of states (PDOS and DOS) analyses (Figure 2c), based on the optimized structure, provide insight into the electronic structure of Zn­(pyrazole–NDI). The molecular orbitals (MOs), which have significant contributions from the p-orbitals of C and O, show prominent DOS just above the Fermi level (*E*_*f*_), highlighting their role as primary e^–^ acceptors during initial reductions. These orbitals are part of the delocalized π-system, formed by the combination of atomic orbitals from C, O, and other atoms in the conjugated linker, resulting in extended molecular orbitals that spread across the entire system. This delocalization allows the e^–^ density to be distributed over the linker and not confined to individual atoms. In contrast, significant N p-orbital contributions appear only at higher energies (∼+5 eV), suggesting their involvement in subsequent reduction steps after the lower-energy molecular orbitals with dominant C/O p-character are occupied. This pattern supports a sequential charge transfer process, where e^–^ initially delocalizes across the NDI core with a clear preference for molecular orbitals with dominant C/O p-character, before progressively enriching N orbitals in later stages. Such behavior reflects stepwise e^–^ transfer between redox-active sites within the linker framework. Meanwhile, Zn d-orbitals contribute minimally near the Fermi level, reinforcing the structural—rather than redox-active—role of Zn nodes. Additionally, the clustering of C, N, and O p-derived states near the Fermi level indicates an accessible manifold of linker-centered frontier states, consistent with partial electronic delocalization within the linker π-system.

Figure 2d shows the calculated electronic band structure (EBS) for neutral Zn­(pyrazole–NDI), with *E*_*f*_ marked by a dashed red line. The bands near the Fermi level exhibit low dispersion, indicating that valence and conduction states are localized on the linkers, which act as redox centers. At the PBE level, the calculated band gap is 1.37 eV, which is consistent with semiconducting behavior, although this value should be interpreted with caution because semilocal GGA functionals are known to underestimate band gaps. To assess this effect, we performed a single-point hybrid-DFT calculation at the PBE0 level (25% exact exchange) on the optimized unit cell using the same fixed geometry employed for the DOS/PDOS analysis. The hybrid calculation yields an indirect band gap of 2.56 eV while preserving the qualitative frontier-state character: the band-edge states remain dominated by ligand-centered C/N/O contributions, whereas Zn d states do not become the primary accepting states. This gap, together with the limited band dispersion, supports the conclusion that electron transport occurs via redox-active sites within the NDI chromophore, where electron density is added and preferentially redistributed to carbonyl and imide moieties, in contrast to band-like transport. Restricted intermolecular interactions, as evidenced by the lack of significant π–π overlap between adjacent linkers, further reinforce this mechanism. Thus, charge transport in this framework cannot originate from extended states but instead arises from dynamically localized redox processes.

### Charge Redistribution and Identification of Active Redox Centers in Zn­(pyrazole–NDI) MOF

Figure 3 summarizes the evolution of charge density in the Zn­(pyrazole–NDI) MOF during sequential reductions, as revealed by AIMD simulations. In panel [Fig fig3]a, the expanded unit cell highlights the periodicity of the crystal lattice. In panel [Fig fig3]b, the charge-density-difference isosurfaces reveal that, after the addition of the first e^–^, the excess charge localizes predominantly in the carbonyl/imide region, with the largest accumulation on carbonyl C/O sites, imide N atoms, and selected C sites within the π-conjugated NDI core. Following the addition of the second e^–^, charge redistribution continues within the same linker-centered manifold, with additional e^–^ density accumulating in the carbonyl-containing region, indicating that these moieties remain key contributors to charge accommodation during progressive reduction. Notably, the π-system of the NDI core also experiences significant enrichment at selected C positions, reflecting a broader delocalization of the added charge across the aromatic framework. Although the N atoms, both within the pyrazole rings and the NDI core, exhibit only a subtle (yet appreciable) increase in e^–^ density during the second reduction, this discernible enrichment supports their involvement and aligns with a multisite reduction mechanism. This behavior is consistent with the quadrupolar nature of the NDI chromophore, where electron-withdrawing imide units attract charge density while the aromatic naphthalene core remains electron-donating. Importantly, sequential charge redistribution of this kind has been experimentally observed in NDI-based systems. In P­(NDI2OD-T2), for example, UV–vis spectroelectrochemistry, NEXAFS, and resonance Raman spectroscopy reveal ground-state bleaching, red shifts in carbonyl stretching modes, attenuation of C–C/C=C and C=O bands, and the emergence of new low-energy vibrational features upon stepwise reduction—direct signatures of polaron and bipolaron formation localized predominantly on NDI carbonyl groups.^66^ These observations provide an experimental analogue to the reduction-induced charge localization and redistribution predicted for the Zn­(pyrazole–NDI) framework. Moreover, the absence of significant charge density at the Zn nodes in both reduction steps, together with their minimal d-orbital contribution to the DOS, supports their role as structural stabilizers rather than redox-active centers.

**Figure 3:**
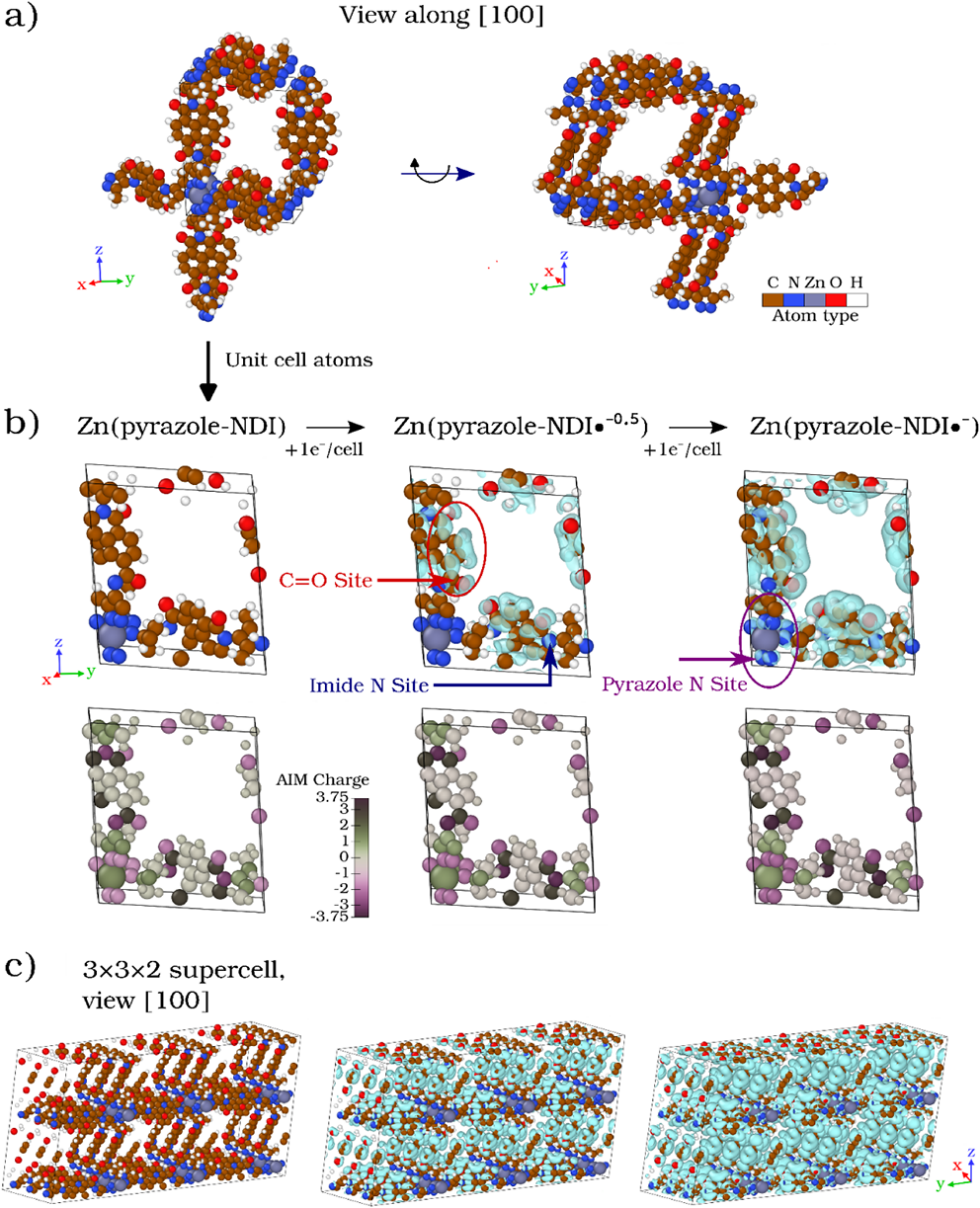
Evolution of charge density in the Zn­(pyrazole–NDI) MOF during successive reduction processes. (a) Expanded unit cell atoms highlighting the periodicity of the crystal lattice, with the right panel showing the structure along the [100] direction. (b) Charge density difference isosurfaces (isovalue = 0.0005 e^–^/Å^3^) and AIM charge maps illustrate the initial localization at C=O sites after the first reduction (with a slight additional density also discernible at imide N atoms), and the emergent density around pyrazole N sites during the second reduction. AIM charges are visualized using the divergent colormap,^67^ in which dark magenta tones denote electron-rich sites (negative AIM charge), dark green tones indicate electron-poor regions (positive AIM charge), and near-neutral atoms appear in muted gray hues. (c) 3 × 3 × 2 supercell along [100], revealing extended charge transport pathways and emphasizing the material’s 3-D connectivity.

The bottom row of panel 3b shows that upon initial reduction (corresponding to the addition of one e^–^ per unit cell) to form Zn­(pyrazole–NDI•^–0.5^), O atoms (initially represented in lighter magenta within the AIM charge color-coding) act as the primary charge acceptors, leading to a significant accumulation of e^–^ density in these regions. For clarity, adding +1 e^–^ per unit cell (containing two NDI linkers) yields this electronic state in which the excess charge dynamically redistributes across both linkers. This redistribution manifests visually as a progressive deepening of magenta intensity at the most electron-rich sites. A discernible increase in e^–^ density also appears at the imide N atoms, observed as further darkening of their already deep magenta tones. At the π-system C atoms, the change is much more subtle, reflected in a shift from a pale greenish-gray hue to a more neutral gray. The last column of panel [Fig fig3]b illustrates the second reduction (corresponding to the addition of two e^–^ per unit cell) to Zn­(pyrazole–NDI•^–^), where O atoms continue to accumulate additional e^–^ density, now reaching the darkest magenta values in the scale. Simultaneously, N atoms in the pyrazole rings exhibit a further increase in electron population, expressed as an additional deepening of their magenta tones relative to that of the first reduction. In contrast, Zn atoms exhibit no significant change in their Bader charge colors, reinforcing their structural role.

In addition, the carbonyl C atoms (C=O) bonded to the imide N atoms in the NDI consistently exhibit a dark green coloration on the AIM charge scale across all of the redox states. This persistent darkness arises from the pronounced polarization experienced by these C atoms, each bonded to two highly electronegative atoms, which leaves them significantly e^–^-deficient and relatively positive. As a result, any subtle changes in e^–^ density on C=O C atoms during successive reductions are not visually apparent on the color scale. Nevertheless, quantitative analysis of AIM charges (Table S1) reveals that these carbonyl C atoms experience a modest, but measurable, accumulation of e^–^ density upon reduction: the difference in AIM charge, *Q*_*C*_(neutral) – *Q*_*C*_ Zn­(pyrazole–NDI•^–^), is −0.0415 e.

These theoretical descriptions reinforce the idea that reduction in the Zn­(pyrazole–NDI) MOF does not occur homogeneously throughout the pyrazole–NDI linker but instead is localized at specific sites. Although the exact atom-resolved Δ*q* values are somewhat sensitive to the chosen exchange-correlation functional, the PBE0 benchmark preserves the same qualitative linker-centered, carbonyl-containing reduction picture. Based on the analysis of electronic redistribution, three classes of redox centers can be distinguished within the redox-active unit: (i) *Primary redox centers* accumulate e^–^ charge from the early stages of reduction. The most notable of these are the imide N atoms (average Δ*q* = −0.0577 e for the first reduction) and the carbonyl C atoms (average Δ*q* = −0.0559 e), which act as the dominant electron-uptake sites, followed by the carbonyl O atoms (average Δ*q* = −0.0214 e). Considering the C=O moiety, the average Δ*q* is −0.0386 e in the first reduction. Additionally, aromatic C atoms in the π-system of the NDI core also participate in the early stages of e^–^ accumulation, with an average Δ*q* of −0.0097 e. In the second reduction, however, the imide N atoms do not gain further electron density (average Δ*q* = +0.1091 e), while the carbonyl C and O atoms continue to accumulate charge (average Δ*q* = −0.0432 e and −0.0308 e, respectively), and the aromatic C atoms display only a minor additional increase in electron density (average Δ*q* = −0.0074 e). (ii) *Dynamically activated redox centers* begin to accumulate e^–^ charge only as the degree of reduction increases; these sites, such as the N atoms in the pyrazole group, remain largely inactive during the initial reduction but become electron-accepting once primary centers are partially filled. For the subset of pyrazole N atoms, the average Δ*q* is −0.0379 e, highlighting their dynamic role, with redox activity triggered in successive reductions. (iii) *Redox-inactive regions* remain unchanged in their e^–^ density throughout the process. In particular, both Zn centers identified display minimal charge variation across both reductions, confirming their negligible participation in redox events. This differentiation in the electronic response of the linker reveals the sequential nature of the reduction mechanism in this system. Crucially, this site-specific charge localization hierarchically activates redox events, thereby defining when and where charge transport can occur. In other words, the availability of hopping pathways depends on the reduction level, which selectively activates or attenuates discrete redox sites that enable charge transfer.

Panel [Fig fig3]c provides a spatial representation of how additional e^–^ introduced during successive reduction steps propagates through the Zn­(pyrazole–NDI) MOF lattice. The charge density isosurfaces, mapped over a 3 × 3 × 2 supercell, reveal a progressive expansion of electronic density throughout the framework as the reduction state increases. Initially, e^–^ charge accumulation is confined to specific redox-active sites, but as reduction progresses, the electronic density expands more uniformly, occupying previously uncharged local interstitial sites within the architecture. This indicates that the material effectively accommodates additional e^–^ while maintaining its structural integrity, emphasizing MOFs’ robustness. The observed charge density propagation across the extended framework highlights the material’s ability to support redox-hopping transport beyond localized molecular sites. Unlike band-like conduction, which requires extended orbital overlap, this mechanism relies on a 3D spatially connected array of electronically coupled redox centers. The uniformity of the electronic charge distribution, despite the nonparallel orientation of the NDI linkers relative to the crystallographic axes, indicates that charge transfer is facilitated through a 3D network. This behavior ensures that the MOF remains electronically active across its entire structure, optimizing the charge mobility within the periodic lattice.

Detailed AIMD simulations and subsequent structural and electronic analyses of the neutral Zn­(pyrazole–NDI) MOF are provided in the Supporting Information. Key results include evidence of structural stability from internal energy equilibration, excellent agreement between simulated and experimental XRD patterns, and precise reproduction of experimental UV–vis spectral signatures (Figures S1 to S3). Collectively, these findings validate the accuracy and reliability of the optimized structure employed throughout this study, which is also provided in the SI.

Additional AIMD simulations of the partially reduced (+1 e^–^ per unit cell, equivalent to +0.5 e^–^ per pyrazole–NDI unit), the singly reduced (+2 e^–^ per unit cell, i.e., +1 e^–^ per pyrazole–NDI unit), and further reduced states (+3 e^–^ and +4 e^–^ per unit cell) are also discussed in the SI. These simulations reveal progressive changes in internal energy and charge redistribution across the NDI ligands, demonstrating the robust stability and efficient charge-accommodation capabilities of Zn­(pyrazole–NDI) MOF under multiple reduction conditions (Figure S1).

### Influence of Redox States on Structural Reorganization and Conductivity

The influence of electronic reduction on the structural dynamics of the Zn­(pyrazole–NDI) framework was assessed by tracking three geometric descriptors during AIMD simulations: (a) The dihedral angle between adjacent NDI linkers along the *y*-axis. (b) The O···O separation between linkers in the nodal plane. (c) The linker–Zn–linker angle at the metal node (Figure 4).

**Figure 4:**
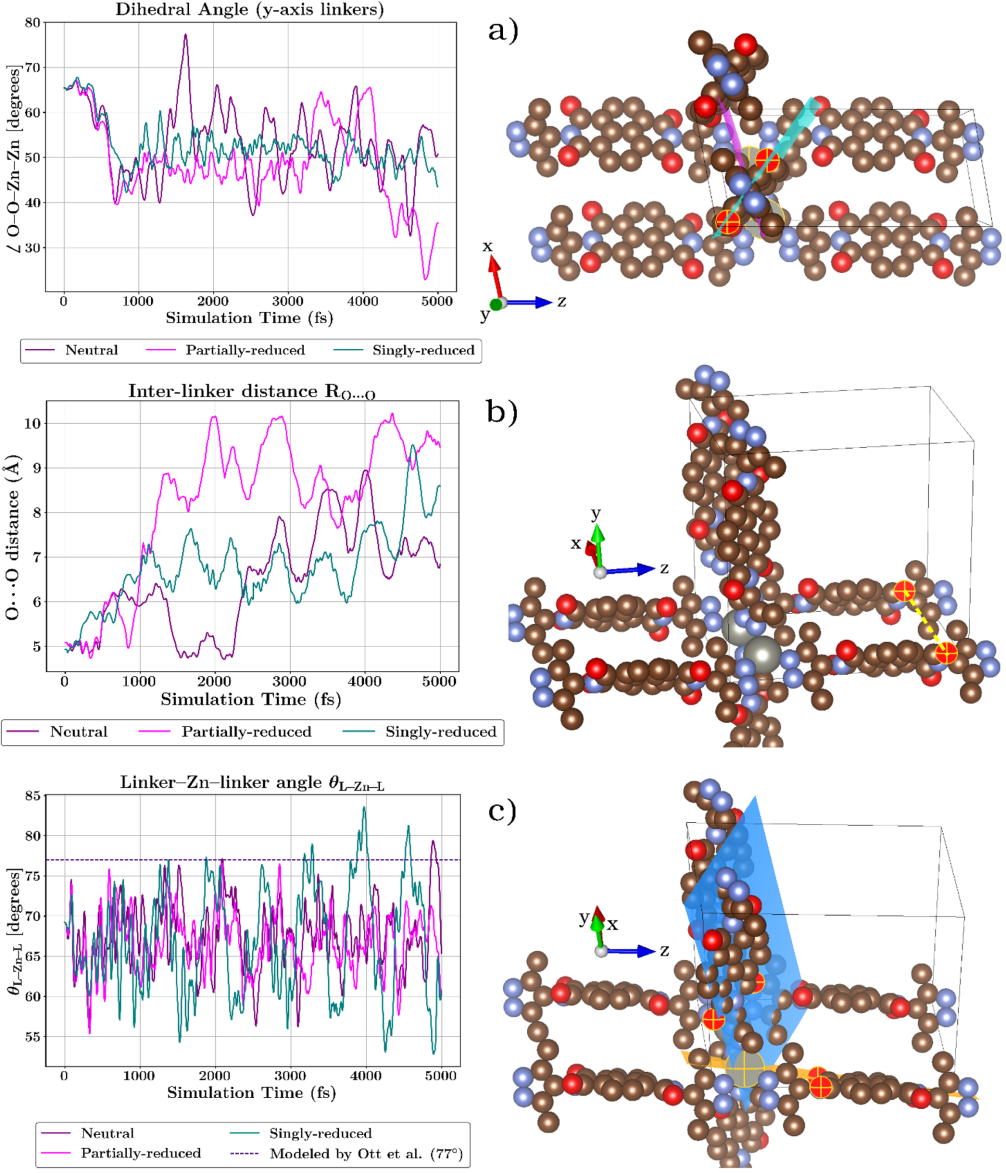
Structural parameters of the Zn­(pyrazole–NDI) MOF tracked during 300 K AIMD simulations for the neutral, partially reduced, and singly reduced states. (a) Dihedral angle ∠O–O–Zn–Zn. Partial reduction (pink) exhibits coordinated minima in the dihedral angle that coincide with maxima in the O···O separation (panel b), yielding a moderate but systematic global correlation (*r* = 0.447). The most prominent cooperative events occur at 4.3–4.4 ps and 4.82–4.84 ps, reflecting Zn-mediated reorientation of orthogonal linkers approaching coplanarity. In contrast, the singly reduced state (green) displays reduced coplanarity, with angles clustering near 50–55°, consistent with localized electronic strain and weakened interlinker communication. (b) Interlinker O···O distance R­(O···O) within the nodal plane. Partial reduction produces the widest excursions (4.7–10.2 Å), with maxima synchronized with dihedral minima in (a), indicating electronically mediated coordination between linkers bound to the same Zn node. (c) Linker–Zn–linker angle θ­(L–Zn–L). The angle between pyrazole–NDI linkers bound to a common Zn node (one oriented along *y*, the other along *z*) fluctuates around the ∼77° reference reported by Ott et al.^34^ Across redox states, mean values remain similar (neutral: 67.6°, partially reduced: 66.7°, singly reduced: 66.9°), but the singly reduced state exhibits a markedly broader distribution (≈53–84°), reflecting additional electronic strain and increased configurational disorder.

Together, these coordinates describe how reduction impacts interlinker alignment and, consequently, redox-mediated charge transport. In the neutral state, the framework explores a wide ensemble of thermally accessible geometries with no persistent alignment motifs, reflecting a structurally flexible but electronically uncorrelated lattice that establishes the baseline from which reduction-induced organization emerges.

Partial reduction, Zn­(pyrazole–NDI•^–0.5^), induces a transient increase in structural coherence: pyrazole–NDI linkers oriented along the *y* direction intermittently adopt more coplanar arrangements and, when sampled at adjacent positions along the *x*-axis, form short O­(carbonyl)···H­(CH_3_–pyrazole) contacts that stabilize these configurations even in the absence of π–π overlap (Figure 4a). This electronically driven prealignment enhances interlinker communication within the redox-active lattice. A quantitative analysis reveals a moderate but systematic correlation (*r* = 0.447) between decreases in the ∠O–O–Zn–Zn dihedral angle and expansions in the O···O separation of orthogonally oriented linkers in the partially reduced state. These coordinated fluctuations occur at well-defined intervals (1.10–1.18 ps, 1.33–1.37 ps, 2.8–3.0 ps, 4.3–4.4 ps, and 4.82–4.84 ps), where dihedral minima coincide with O···O maxima, indicating Zn-mediated electronic coupling between motions occurring along perpendicular crystallographic directions. These cooperative events reflect a charge-mediated structural response in which enhanced coplanarity of the π-system of the *y*-oriented linker with the *xy* plane is systematically accompanied by increased alignment of the π-system of the *z*-oriented linker with the *xz* nodal plane (as observed in panels [Fig fig4]a and b).

In contrast, the singly reduced state introduces a localized electronic strain. Bond-length contractions within the pyrazole unit and reorganization around the Zn node displace peripheral groups and weaken the noncovalent interactions that stabilize the partially reduced state. Linkers positioned along orthogonal crystallographic directions consequently move farther apart, adopt larger dihedral angles, and reduce the coherence of the charge-transport network. In this regime, no correlation is observed between the O···O maxima and dihedral minima, revealing weakened electronic communication between orthogonal linkers and indicating that the structural response becomes strain-limited rather than electronically coordinated. Short-range O­(carbonyl)···H­(CH_3_–pyrazole) interactions continue to appear even at large O···O separations, suggesting that these weak contacts impose a lower bound on how closely orthogonal linkers can approach despite accumulating electronic strain. Charge accumulation on carbonyl groups also enhances the repulsion of O···O between linkers bound to the same Zn node, and this (together with the rigidity of the tetrahedral Zn environment) prevents orthogonal NDI units from adopting electronically favorable orientations.

The impact of this structural adjustment on e^–^ transport is further corroborated by the results in Figure 5a, which illustrate the dependence of the electron transfer coupling energy on the relative orientation of neighboring π-systems along the [010] direction. As the dihedral angle between adjacent π-systems decreases (ϕ­(π_1_ – π_2_) → 0), the electronic coupling energy increases, reinforcing the notion that parallel linker configurations enhance electronic interactions and facilitate charge transport through the framework. Moreover, the electronic band structure analysis provides additional evidence supporting an increased conductivity in the partially reduced state. A marked increase in band dispersion near the Fermi level in Zn­(pyrazole–NDI•^–0.5^) (Figure 5b) points to an enhancement in electronic delocalization across the redox centers. The emergence of more undulated bands further supports this interpretation. Additionally, the near closure of the calculated band gap (0.0002 eV) in this state indicates a low-gap mixed-valence regime with enhanced electronic coupling between redox-active linkers. In this regime, redox hopping remains the dominant transport mechanism but with significantly reduced energetic barriers for charge transfer.

**Figure 5:**
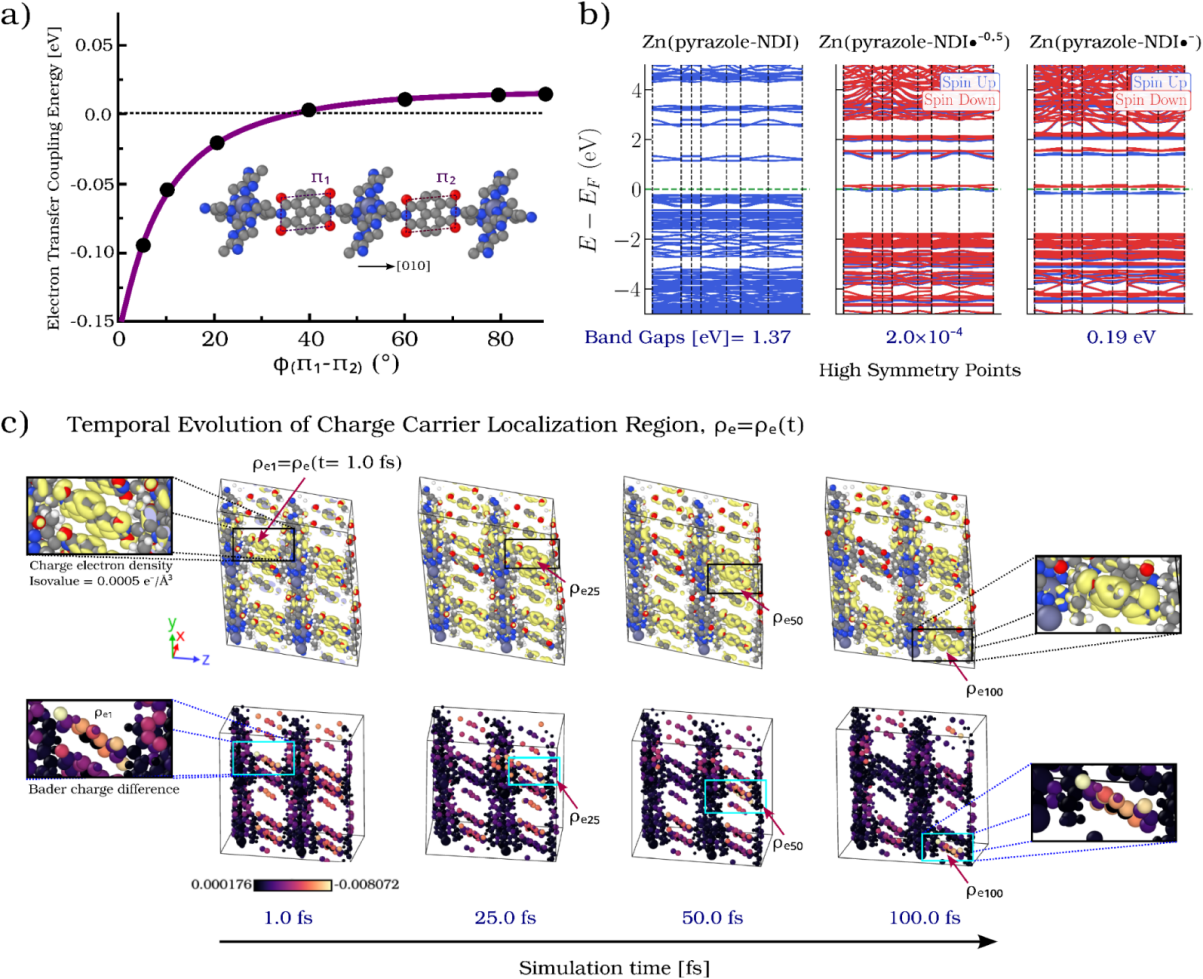
(a) Electron Transfer Coupling Energy as a function of the dihedral angle ϕ­(π_1_ – π_2_) in the Zn­(pyrazole–NDI) MOF, illustrating how relative linker orientation modulates electronic communication between adjacent π-systems (full computational details are provided in SI). (b) Electronic band structures of Zn­(pyrazole–NDI) in three redox states: neutral (Zn­(pyrazole–NDI), left), partially reduced (Zn­(pyrazole–NDI•^–0.5^), center), and singly reduced (Zn­(pyrazole–NDI•^–^), right). The neutral state exhibits a band gap of 1.38 eV. In the partially reduced state, the gap nearly vanishes (2.0 × 10^–4^ eV), suggesting quasi-metallic behavior, whereas in the singly reduced state, a small but finite gap reopens (0.19 eV), indicating decreased electronic coupling. Spin-polarized calculations define the gap as the smallest energy separation between occupied and unoccupied states across spin channels. The green dashed line marks *E*_*f*_. The band structures were computed from snapshots taken at 5000 fs of the AIMD trajectories shown in Figure 4. (c) Temporal evolution of charge-carrier localization in a 2 × 2 × 2 Zn­(pyrazole–NDI) MOF supercell during an AIMD simulation (+1 e^–^ per supercell). Yellow isosurfaces (top row, isovalue = 0.0005 e^–^/Å^3^) depict the additional electron’s charge density difference, initially localized at ρ_e1_ (1 fs) in the top-back-left linker. The carrier migrates to linkers coordinated to the same Zn node: along the *z*-axis (ρ_e25_), along the −*x* direction (ρ_e50_), and finally along the −*y* direction (ρ_e100_, 100 fs). Bader charge-difference maps (bottom row) confirm charge redistribution, highlighting regions of accumulation (yellow) and depletion (purple/black). The carrier avoids linkers aligned along the *y* direction, emphasizing preferential transport via *z*-aligned linkers and a redox-hopping mechanism mediated by Zn-node connectivity. This dynamic behavior emphasizes the potential of this MOF for efficient three-dimensional charge transport.

Conversely, Figure 5b shows a band gap of 0.19 eV for the Zn­(pyrazole–NDI•^–^) state, diverging from the near-gap-closure regime obtained for the Zn­(pyrazole–NDI•^–0.5^) state. Experimentally, a decrease in conductivity has also been reported when the system reaches the Zn­(pyrazole–NDI•^–^) state, corresponding to +1 e^–^/linker or +2 e^–^/unit cell, compared to the Zn­(pyrazole–NDI•^–0.5^) state (*x* = 0.5, equivalent to +0.5 e^–^/linker or +1 e^–^/unit cell). Here, *x* follows the experimental convention and denotes the mole fraction of e^–^ with respect to the NDI linkers. In our simulations, the redox composition is defined by the number of electrons added per periodic unit cell because each unit cell contains two pyrazole–NDI linkers, +1 e^–^ and +2 e^–^ per unit cell correspond to *x* ≈ 0.5 and *x* ≈ 1.0, respectively, in the experimental convention. In general, redox conductivity in Zn­(pyrazole–NDI) MOF increases by a factor of 10^4^ in the mixed-redox state, *x* = 0.5 or 1.5, compared to states *x* = 1.0 or 2.0. Ott et al.^34^ proposed that when 1 e^–^/unit cell is introduced (*x* = 0.5), approximately half of the NDI linkers are reduced to NDI•^–^, maximizing redox conductivity in the range of 10^–5^ to 10^–6^ S·cm^–1^.

While the interpretation put forth by Ott et al.^34^ is useful for qualitatively rationalizing the conductivity increase based on the number of vacant redox sites, our calculations provide additional physical insights into the transport mechanism. Our results show that upon adding 1 e^–^/unit cell, the additional e^–^ is not localized on a single linker but is delocalized across both linkers within the unit cell, as shown in Figure 3. Furthermore, in successive reductions (*x* = 1.5, 2.0), added e^–^ continues to exhibit this delocalization behavior. It is important to note, however, that the fully symmetric delocalization predicted by periodic DFT represents the idealized limit of a pristine unit cell. The discrepancy between the calculated partially reduced UV–vis spectrum (Figure S3) and the experimental trace^34^ at *x* = 0.5 further suggests that the mixed-valence signatures observed in real films necessarily arise from a symmetry-breaking agent, consistent with the strong counterion-dependent conductivity and activation barriers reported by Ott et al.

From our perspective, the increase in conductivity is not solely due to a 1:1 ratio between e^–^ in the MOF lattice and the available redox centers. In contrast to a purely statistical argument, our interpretation follows a structure-driven analysis in which electron distribution-induced structural reorganization enhances the probability of redox hopping and optimizes charge transport. In other words, the relative structural reorganization induced by increased electron density at specific redox centers modifies electronic coupling between redox sites, thereby optimizing the overall conductivity in the MOF lattice. These calculations reveal that the increase in electronic density at specific redox center atoms following the reduction of the Zn­(pyrazole–NDI) MOF significantly influences the spatial configuration of the pyrazole–NDI ligands. This reorganization enhances electronic coupling in certain cases, optimizing ET and consequently improving the material’s electrical conductivity. As shown, maximum conductivity is achieved when the added e^–^ are equally delocalized over both linkers in the unit cell, with each carrying a −0.5 e^–^ charge, thus enabling optimal charge transport. What this implies is that maximal conductivity does not arise from electron count alone but from a dynamically stabilized mixed-valence configuration that enhances electronic coupling between redox sites. Within the intrinsic framework response discussed here, the structural-reorganization and redox-site-coupling effects inferred from our calculations should be viewed as experimentally testable mechanistic predictions arising from electron-distribution-induced local geometric reorganization. In particular, operando IR/Raman,^68^ UV–vis–NIR,^69^ and EPR spectroelectrochemical measurements^70^ across the relevant redox states would be especially valuable for probing the proposed local geometric reorganization, symmetry breaking, and enhanced electronic coupling between redox sites in the partially reduced (mixed-valence) regime.

Figure 4c shows the evolution of the angle between the two pyrazole–NDI linkers bound to the same Zn node (one oriented along the *y*-axis and the other along the *z*-axis) with the reference value of 77.0° from Ott et al.^34^ included for comparison. Across all redox states, the angle samples a broad range of thermally accessible configurations (neutral: 55.9–79.4°, partially reduced: 55.3–76.5°, singly reduced: 52.8–83.6°), with similar average values (67.6°, 66.7°, and 66.9°, respectively). The neutral and partially reduced states exhibit comparable behavior, indicating that the first added electron does not trigger a systematic reorganization of this structural descriptor. In contrast, the singly reduced state displays a noticeably broader angular dispersion, reflecting the electronic strain associated with the additional charge localized near the pyrazole N atoms bound to Zn. This increased flexibility points to incipient structural disorder around the node, which may hinder efficient charge transfer and contribute to the reduced conductivity in the singly reduced regime.

Overall, these trends reveal a clear structure–transport relationship: partial reduction promotes redox connectivity through coordinated, electronically mediated structural motions, whereas deeper reduction disrupts this alignment and reduces e^–^ mobility. A detailed analysis of these structural descriptors, including full angle distributions, time-resolved trajectories, and distance-angle correlations, is provided in the Supporting Information.

### Electron Transport in MOF Architectures

Figure 5c illustrates the time-resolved evolution of the excess charge localization region (ρ_e_ = *f*(*t*)) during an AIMD simulation of a 2 × 2 × 2 supercell of the Zn­(pyrazole–NDI) MOF with one added electron. By tracking the charge density as a function of time, we observe that although the excess e^–^ remains partially delocalized over the framework, a finite region of concentrated e^–^ density can be identified, whose position shifts discretely between neighboring NDI linkers. Consistent with this picture, from the beginning of the simulation the e^–^ density (top) and the corresponding Bader charge difference (bottom) display a dominant contribution on a specific NDI linker while retaining partial delocalization over other linkers within the supercell. This spontaneous partial delocalization, even without an applied electric field, reflects the system’s intrinsic conductive nature that arises from the unique bonding network and orbital arrangement of the Zn­(pyrazole–NDI) MOF described above. Throughout the simulation, the charge carrier localization region shifts between linkers connected to the same Zn node at each stage. The initial charge density, predominantly localized on a linker positioned in the upper back-left corner of the supercell (ρ_e1_, *t* = 1 fs), migrates first to an adjacent linker along the *z*-axis (ρ_e25_, *t* = 25 fs), then to a linker in the −*x* direction (ρ_e50_, *t* = 50 fs), and finally localizes on a linker positioned along the −*y* direction (ρ_e100_, *t* = 100 fs). Bader charge difference maps corroborate this behavior, revealing regions of charge accumulation (yellow) and depletion (black) at different time points. After 100 fs, the charge carrier localization region (ρ_e100_) is entirely displaced compared to its initial position (ρ_e1_), facilitated by the connectivity provided by the Zn nodes.

These results demonstrate that the charge carrier’s motion is not confined to a single plane but involves multidirectional hopping within the three-dimensional framework. The Zn node plays a critical role as an electronic mediator, functionally connecting linkers oriented in different directions and enabling efficient three-dimensional charge transport. An intriguing observation is the absence of charge carrier localization on linkers oriented along the *y*-axis throughout the simulation. This behavior contrasts with the clear preference of the additional e^–^ to move between linkers aligned along the *z*-axis. This may be because, as shown in our calculations discussed above, the electronic coupling between adjacent linkers is stronger along the *z*-axis direction than in the *xy*-plane. The coordinative architecture of the Zn node likely contributes to this exclusion, acting as a more efficient mediator for charge transfer along preferential directions within the MOF’s three-dimensional framework.

Although the charge carrier exhibits partial delocalization from the beginning of the simulation, its trajectory appears to prioritize a strategic three-dimensional path among linkers connected to the same Zn node. This pattern is not purely random; the movement between the *z*, *x*, and *y* directions may be influenced by the specific orientation of the pyrazole–NDI linkers and their ability to stabilize electronic transitions by minimizing local electronic repulsion.

This analysis confirms that charge transport in this MOF follows a redox-hopping mechanism in which e^–^ is transferred between spatially separated pyrazole–NDI linkers that act as active redox centers. Adjacent linkers involved in a two-link redox-hopping event are coordinated to the same pair of Zn^2+^ metal nodes but are not in direct contact. This behavior is similar to that observed in other MOFs,^50,71–74^ where redox hopping has been suggested to occur between active sites associated with organic linkers.

### Ion Effects on Redox Conductivity and Electron-Hopping Mechanisms in MOFs

#### Ion-Mediated Coordination Dynamics and Structural Reorganization

The presence of electrolyte in redox-active MOFs has been experimentally shown to influence charge transport by affecting the energetic landscape and the mobility of charge carriers.^34,50,62–64^

To elucidate this effect at the atomistic level, we computed two-dimensional potentials of mean force (PMFs) trajectories describing local K^+^ motion within the Zn­(pyrazole–NDI) framework, as a function of the smooth coordination number (CN) defined by the distances between K^+^ and O + N donor atoms and the third-shortest K–O distance (d_3_) (Figure 6a,b). These PMFs reveal a sequence of metastable coordination states that quantify the ionic and structural reorganization barriers (Δ*G* ^‡^) obtained directly from AIMD trajectories, which were correlated with intrinsic e^–^-hopping barriers (Δ*G* ^‡^_IEH_) derived from Marcus–CDFT analysis (Figure 7). In this work, we distinguish two complementary reorganization processes: (i) The PMF barriers (Δ*G* ^‡^), obtained from AIMD, describe the ionic and lattice rearrangements associated with K^+^ motion through coordination states Ia–IIIa, which define the structural precursors for subsequent electron-transfer analysis (Figure 7). (ii) The intrinsic electron-hopping parameters (λ_in_, Δ*G*, and Δ*G* ^‡^_IEH_), obtained from Marcus–CDFT, quantify the internal reorganization energy (λ_in_) and the corresponding activation barrier for charge transfer (Δ*G* ^‡^_IEH_) between adjacent NDI linkers within a given coordination basin. Thus, λ_in_ and Δ*G* ^‡^_IEH_ refer exclusively to the structural relaxation and barrier associated with the e^–^ hopping event itself and not to the ionic reorganization between coordination states, which is captured by the PMF analysis.

**Figure 6:**
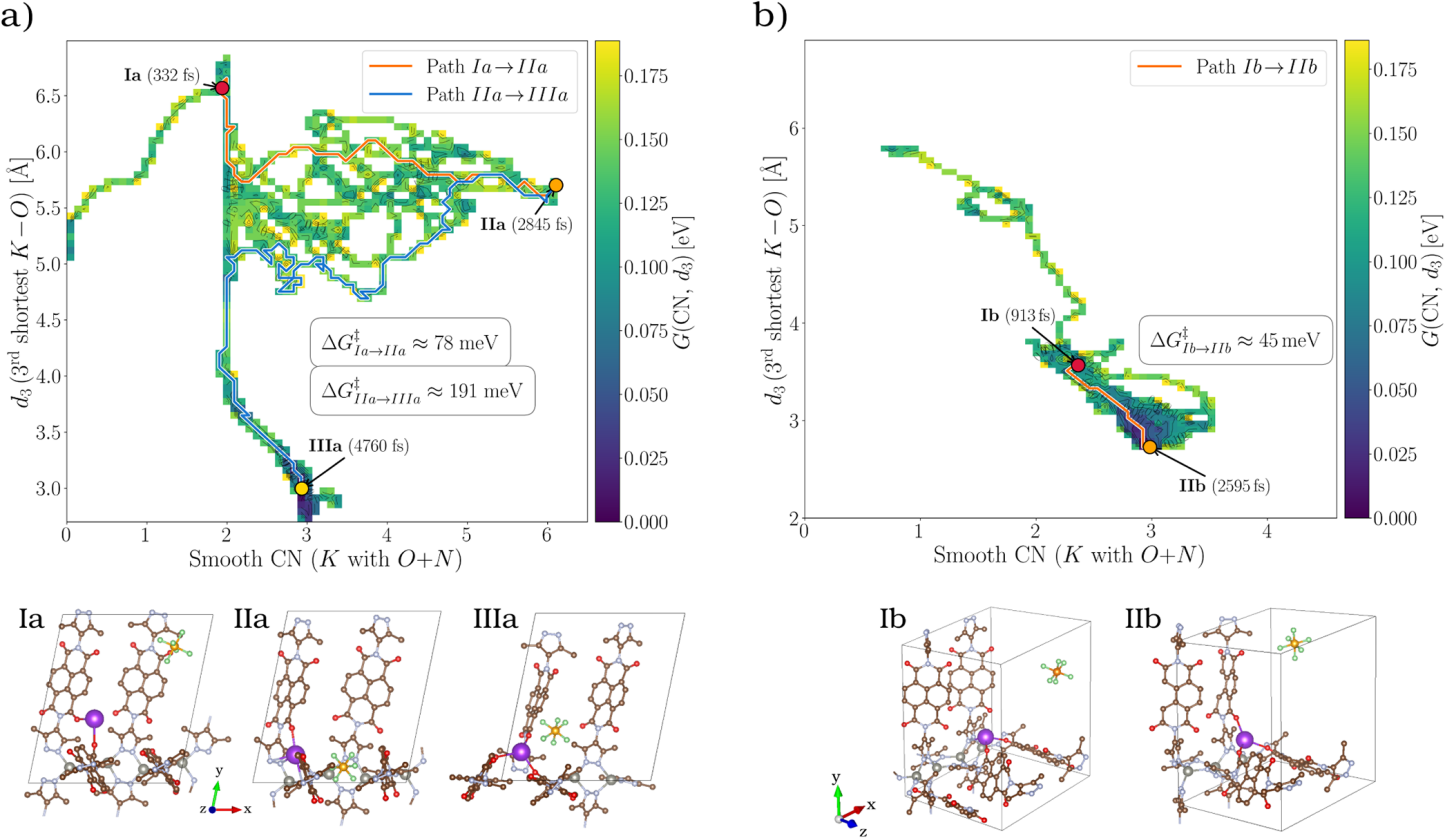
(a,b) Two-dimensional potentials of mean force (PMFs) describing the free-energy landscape of K^+^ motion in the Zn­(pyrazole–NDI) framework as a function of the smooth coordination number (CN) between K^+^ and the O + N donor atoms and the third-shortest K–O distance (d_3_, left vertical axis). Here, Smooth CN denotes a continuous, distance-weighted coordination number between K^+^ and surrounding O and N donor atoms, evaluated using a Fermi-type switching function with cutoff distances *r*_*c*_ = 3.48 Å for both K–O and K–N interactions. The right vertical axis reports the PMF free-energy scale, *G*(CN, d_3_), encoded by the color bar (in eV). The colored background represents the phase space sampled by the AIMD trajectory in this collective-variable space, including repeated subbarrier excursions between neighboring basins. In the neutral lattice (a, 332 K), three metastable basins (Ia–IIIa) correspond to outer-sphere κ^2^-O,O′, mixed κ^2^-O,O′ + κ^4^-N,N′,N″,N‴(pyrazole), and tridentate κ^3^-O,O′,O″ motifs, connected sequentially by Path Ia → IIa (orange, Δ*G* ^‡^ ≈ 78 meV) and Path IIa → IIIa (blue, Δ*G* ^‡^ ≈ 191 meV). In the partially reduced lattice (b, 326 K), the surface collapses to a single accessible Path Ib → IbI (orange, Δ*G* ^‡^ ≈ 45 meV), reflecting enhanced K^+^ mobility and stabilization of the tridentate geometry upon reduction. Representative AIMD snapshots of each coordination state are shown below.

**Figure 7:**
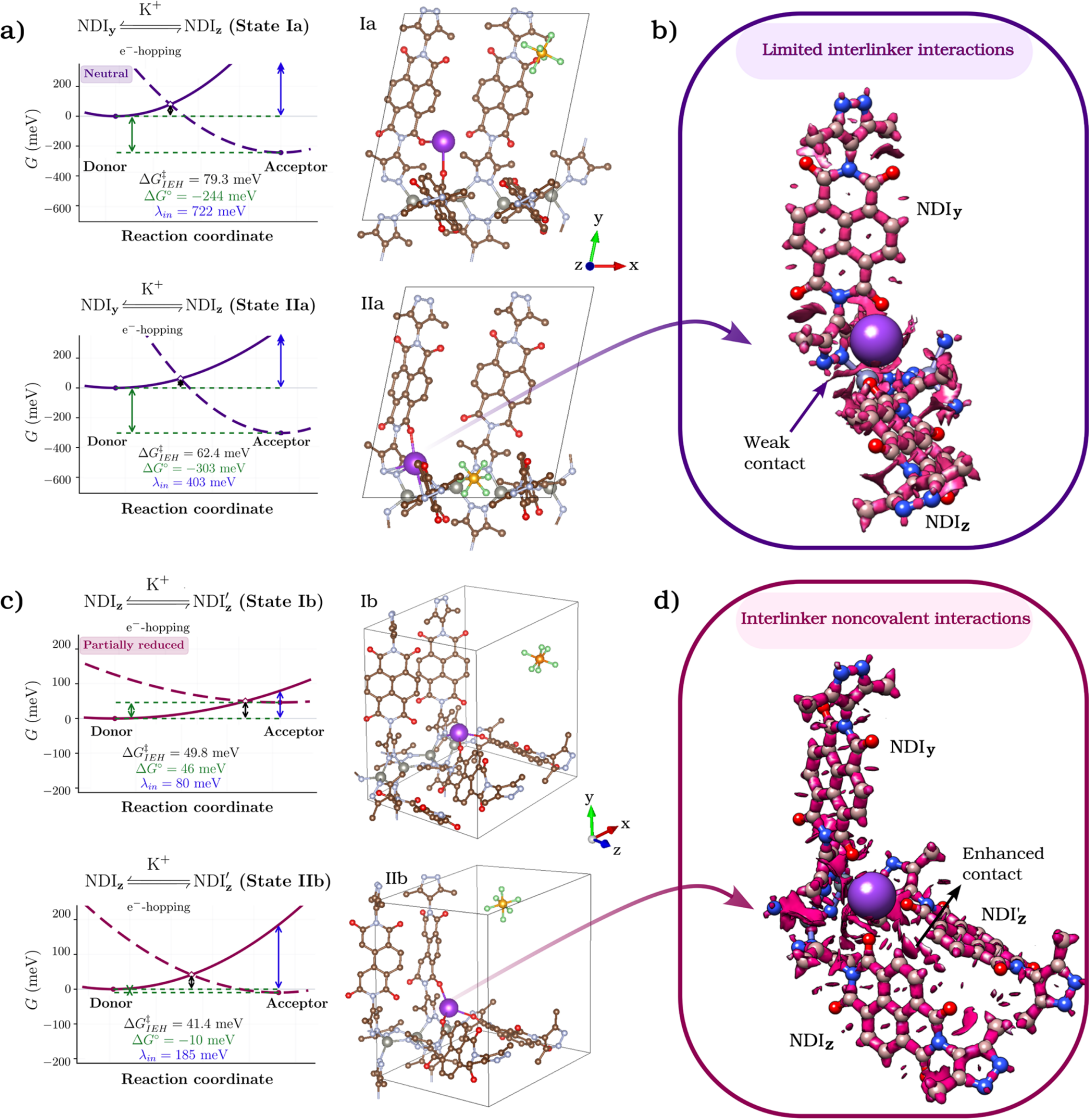
(a,c) Gibbs free-energy surfaces for e^–^ transfer between neighboring pyrazole–NDI linkers obtained from Marcus–CDFT analysis. K^+^ coordination substantially reduces both the internal reorganization energy and the activation barrier for e^–^ hopping, from λ_in_ = 722 meV and, Δ*G* ^‡^_IEH_ = 79 meV in the outer-sphere State Ia to λ_in_ = 403 meV and, Δ*G* ^‡^_IEH_ = 62 meV in the inner-sphere State IIa (panel a), evidencing electrostatic preorganization of donor–acceptor geometries induced by K^+^ binding. Upon partial reduction (panel c), the free-energy profiles further flatten (λ_in_ = 80–185 meV; Δ*G* ^‡^_IEH_ = 41–50 meV), defining a low-barrier regime in which ionic rearrangements and electronic transfer evolve quasi-adiabatically on a coupled free-energy surface. (b) Interaction Region Indicator (IRI) isosurface (isovalue = 1.2) for the local K^+^ coordination environment in the inner-sphere State IIa of the neutral framework. The IRI analysis reveals weak interlinker noncovalent contacts between the pyrazole ring of a *y*-aligned linker and the carbonyl group (C=O) of a neighboring *z*-aligned linker. These weak pyrazole···C=O interactions afford only limited stabilization of the donor–acceptor geometry and do not establish optimal interlinker coupling. (d) IRI isosurface for the local K^+^ coordination environment in the partially reduced State IIb, where a K^+^-bridged trimeric pyrazole–NDI assembly has formed. In contrast to State IIa, the IRI surface reveals a cooperative K^+^-mediated coordination motif involving multiple carbonyl groups, together with additional C–H···π and C=O···H contacts between adjacent NDI units along the *z* direction. This cooperative coordination motif provides enhanced structural stabilization of the trimeric node and is correlated with the low-barrier redox-hopping regime identified for the partially reduced state by the Marcus–CDFT analysis.

#### Ion-Coupled Coordination in the Neutral Framework

Three well-defined coordination basins are identified in Figure 6a. State Ia is an outer-sphere κ^2^-O,O′ coordination involving two carbonyl oxygens from NDI linkers oriented along the *y* and *z* directions. State IIa is an inner-sphere κ^2^-O,O′ + κ^4^-N,N′,N″,N‴(pyrazole) environment with mixed O_2_N_4_ binding. Finally, state IIIa is a tridentate κ^3^-O,O′,O″ configuration in which K^+^ binds three carbonyl oxygens from neighboring linkers, forming a transient trimeric node.

Transitions occur sequentially (Figure 6a, top panel Ia–IIIa) with Δ*G* ^‡^_Ia→IIa_ ≈ 78 meV indicating that the outer-sphere → inner-sphere rearrangement is relatively facile. In contrast, the formation of the tridentate species with Δ*G* ^‡^_IIa→IIIa_ ≈ 191 meV requires significant lattice reorganization. State Ia serves as the precursor for ion migration, defined by weak binding to two carbonyl oxygens (K–O = 3.01, 2.71 Å).

The nearest Zn centers (6.84 Å) confirm that K^+^ lies outside the inorganic node, while secondary donor distances, K–N (pyrazole) = 5.46–5.71 Å and K–N­(imide) ≈ 5.05 Å, exclude any K–N interaction. Marcus–CDFT calculations (Figure 7a) yield λ_in_ = 722 meV and Δ*G* ^‡IEH^ ≈ 79 meV for e^–^ hopping between adjacent NDI linkers, in sharp contrast to the K^+^-free lattice, where λ_in_ = 2384.5 meV and Δ*G* ^‡^_IEH_ = 154 meV. This corresponds to a more than 3-fold reduction in the internal reorganization energy and a reduction of the activation barrier by nearly 50%, showing that K^+^ electrostatically preorganizes the carbonyl environment, stabilizes donor and acceptor geometries, and lowers the intrinsic structural cost of charge transfer through a more adiabatic hopping pathway.

We now turn to State IIa, the inner-sphere κ^2^-O,O′ + κ^4^-N,N′,N″,N‴(pyrazole) coordination environment. Upon transition, K^+^ moves inward to form a mixed O_2_N_4_ inner-sphere environment. Two carbonyl oxygens (K–O = 2.66, 2.73 Å) and four pyrazole Ns from orthogonal linkers coordinate simultaneously: for the *y*-oriented linker, K–N­(pz) = 2.91, 2.91 Å (symmetric); for the *z*-oriented linker, 2.91, 2.88 Å (slightly asymmetric). This κ^2^-O,O′ + κ^4^-N,N′,N″,N‴(pyrazole) geometry defines a quasi-tetrahedral O_2_(N=N)_2_ coordination environment, where the two pyrazole N=N fragments act as bidentate donors that complement the carbonyl oxygens. The K^+^ center is displaced toward the π-rich N=N plane, yielding an anisotropic but electronically delocalized pocket that bridges the *y*- and *z*-oriented linkers. The average K–Zn distance shortens to 4.08 Å (from 6.84 Å), while K–N (imide) ≈ 3.83 Å remains electrostatic. Marcus–CDFT analysis (Figure 7a, State IIa free-energy surface) yields λ_in_ = 404 meV and Δ*G* ^‡^_IEH_ ≈ 62 meV, indicating that a δ­(K^+^)−π­(pyrazole) interaction electrostatically couples the π-systems of two neighboring NDI linkers. This cation-bridged interaction lowers the internal reorganization energy and aligns donor–acceptor orbitals across the *y*–*z* linkers, promoting enhanced interlinker e^–^ transfer.

Finally, in State IIIa, the system adopts an inner-sphere κ^3^-O,O′,O″ coordination environment. In its most stable configuration, K^+^ binds three carbonyl oxygens (K–O = 3.00, 2.78, 2.54 Å) from neighboring NDI linkers, two of which are collinear along the *z* direction, forming transient K^+^-bridged (pyrazole–NDI)_3_ trimers that constitute the structural global PMF minimum. The Zn–O_3_ plane deviates slightly from coplanarity (160.4°), forming a distorted trigonal geometry. N–K^+^ interactions vanish (Zn–N­(pyrazole) = 4.49–4.85 Å; K–N­(imide) ≈ 4.71 Å), while K–Zn = 5.44 Å confirms full embedding in the organic sublattice. The large Δ*G* ^‡^_IIa→IIIa_ ≈ 191 meV arises from the cooperative structural rearrangement required for K^+^ to transition from the highly coordinated O_2_N_4_ environment of State II to the oxygen-dominated κ^3^-O,O′,O″ geometry of State IIIa. This process involves the loss of K–N­(pyrazole) interactions and the simultaneous formation of a new K–O­(carbonyl) bond, driving the alignment of neighboring NDI carbonyls along the *z* direction and yielding a transient K^+^-bridged (pyrazole–NDI)_3_ trimeric motif that preorganizes linkers for e^–^ hopping in the reduced states.

The sequence κ^2^-O,O′ → (κ^2^-O,O′ + κ^4^-N,N′,N″,N‴) → κ^3^-O,O′,O″ captures the gradual internalization of K^+^ from a weakly bound outer-sphere species to a deeply embedded, electronically coupled center. This progression transforms K^+^ from a passive spectator into an active structural mediator, prealigning redox-active linkers for charge delocalization.

Although the PMF barrier Δ*G* ^‡^_IIa→IIIa_ ≈ 191 meV reflects the cooperative lattice reorganization needed to form this tridentate motif, the intrinsic electron-hopping barrier Δ*G* ^‡^_IEH_ ≈ 62 meV is considerably lower. This distinction emphasizes that the PMF barrier tracks large-scale K^+^ migration, whereas Δ*G* ^‡^_IEH_ arises from local relaxation within the donor–acceptor pair environment. Once formed, K^+^-bridged trimers efficiently couple adjacent NDI π-systems, serving as preorganized conduits for charge transfer.

#### Ion-Coupled Coordination in the Partially Reduced Framework

In the partially reduced lattice, the PMF landscape collapses to a single Ib → IIb transition with a smaller reorganization barrier (Δ*G* ^‡^_Ib→IIb_ ≈ 45 meV; Figure 6b), reflecting the softening of the coordination potential upon 1 e^–^/unit cell reduction. The tridentate basin observed in the neutral framework merges with the bidentate state, indicating that K^+^ insertion and charge delocalization proceed cooperatively.

The first coordination configuration, State Ib, corresponds to an inner-sphere κ^2^ −O,O′ arrangement along *z*-oriented linkers. Here, K^+^ binds two carbonyl oxygens (K–O = 2.55, 2.70 Å) from NDI linkers coaligned along z, with a third −C=O oxygen acting as a weak spectator (3.57 Å). The K–Zn distance decreases to 4.92 Å, evidencing deeper insertion relative to the neutral system (6.84 Å). Auxiliary contacts with pyrazole nitrogens [K–N­(pz) = 3.39, 4.22 Å] provide secondary electrostatic stabilization. The two NDI cores forming the κ^2^-O,O′ motif are parallel and collinear (*z*-axis), with a minimum interlinker C···C separation of 4.2 Å and an interplanar angle of ≈78.7°, indicative of stronger electronic coupling. Marcus–CDFT results (Figure 7c, State Ib free-energy surface) yield λ_in_ ≈ 80 meV and Δ*G* ^‡^_IEH_ ≈ 50 meV for e^–^-hopping along *z*–*z* linkers, corresponding to an almost order-of-magnitude reduction in λ_in_ and a 3-fold decrease in Δ*G* ^‡^_IEH_ relative to the K^+^-free system. Partial reduction thus stabilizes the K^+^–linker assembly electronically and enhances the through-space coupling between redox-active NDI units.

The next configuration, State IIb, corresponds to a tridentate κ^3^-O,O′,O″ environment stabilized by noncovalent interlinker interactions. Upon partial reduction, K^+^ overcomes a small reorganization barrier (Δ*G* ^‡^_Ib→IIb_ ≈ 45 meV) to adopt a κ^3^-O,O′,O″ coordination involving three carbonyl oxygens from NDI linkers, two collinear along *z* and one oriented along *y*. The K–O distances (2.55–2.70 Å) define a nearly planar O_3_ coordination pocket that remains dynamically stable throughout the simulation. For this partially reduced State IIb, an Interaction Region Indicator (IRI) analysis (Figure 7d) reveals that subtle noncovalent interlinker interactions originate already in the bidentate configuration and become reinforced upon tridentate K^+^ coordination. Specifically, short C­(π-naphthalene–NDI_1_–*z*)···H–C­(π-naphthalene–NDI_2_–*z*) contacts (3.64 and 3.48 Å) and a weak C=O···H interaction (2.86 Å) appear at the interface between neighboring linkers. These C–H···π and C=O···H contacts bring the π-cores closer together, promoting cooperative polarization and enhanced through-space electronic communication across the K^+^-bridged NDI pair. These noncovalent interlinker interactions directly contribute to lowering the intrinsic electron-hopping barriers in the reduced framework in the presence of K^+^. Consistent with this picture, Marcus–CDFT analysis for this tridentate configuration (Figure 7c, State IIb free-energy surface) yields λ_in_ = 185 meV and Δ*G* ^‡^_IEH_ ≈ 41 meV, establishing K^+^-bridged trimeric motifs as the most kinetically favorable electron-hopping states among the studied configurations. The markedly reduced λ_in_ reflects the strong electrostatic preorganization and cooperative stabilization imparted by K^+^ together with the reinforcing network of interlinker C–H···π and C=O···H contacts. By contrast, the neutral inner-sphere State IIa exhibits only limited direct linker–linker interactions, as shown by the IRI analysis (Figure 7b), consistent with the higher electron-hopping barriers obtained from Marcus–CDFT for the neutral framework (Figure 7a). Once formed, this κ^3^-O,O′,O″ motif gives rise to transient K^+^-bridged (pyrazole–NDI)_3_ trimeric nodes that remain stable over time, serving as structural nuclei for long-range charge delocalization throughout the reduced framework. These K^+^-mediated trimeric motifs should also be viewed as experimentally testable mechanistic predictions. In particular, operando scattering methods^75^ would be especially valuable for probing local, nonperiodic structural correlations associated with transient trimer formation, such as changes in K–O and O···O distances that may not be visible in conventional diffraction. Complementarily, operando X-ray absorption spectroscopy (XAS/EXAFS)^76^ could help assess whether the local coordination environment of the cation evolves in a manner consistent with multidentate carbonyl binding and trimer-mediated bridging between redox-active linkers.

Partial reduction effectively prealigns the carbonyl-rich linkers along the *z*-axis, lowering the entropic cost of K^+^ migration and enabling its full incorporation into the tridentate coordination pocket. The pronounced decrease in the intrinsic electronic reorganization energy (λ_in_) and intrinsic e^–^-hopping barrier (Δ*G* ^‡^_IEH_), for the bidentate and tridentate motifs of the partially reduced lattice arises from this specific K^+^-mediated coordination, which enforces spatial proximity and relative alignment of *z*-oriented NDI linkers, reduces nuclear reorganization within the donor–acceptor pair, strengthens interlinker electronic communication, and facilitates through-space electron transfer between adjacent redox centers. K^+^ thus acts simultaneously as an electrostatic anchor and a redox mediator, stabilizing the κ^3^-O,O′,O″ geometry and enhancing NDI–NDI coupling.

The close correspondence between the ionic coordination barrier obtained from the PMF (Δ*G* ^‡^_Ib→IIb_ ≈ 45 meV) and the electronic hopping from Marcus–CDFT analysis (Δ*G* ^‡^_IEH_ ≈ 41–50 meV) barrier reveals that ionic rearrangement and electron transfer evolve on comparable free-energy scales, establishing an adiabatically gated, strongly coupled ion–electron hopping regime. In this regime, K^+^ migration no longer precedes charge transfer but codrives it, converting local coordination dynamics into an efficient channel for long-range charge propagation. Such cooperative ion–electron coupling—initiated at bidentate motifs and reinforced upon tridentate formation —constitutes the microscopic origin of the enhanced redox conductivity observed experimentally^34^ in the Zn­(pyrazole–NDI) framework.

This ion-gated redox-hopping mechanism is consistent with the low-energy-barrier hopping transport reported in related ion-conducting frameworks, where reduced activation energies are associated with discrete ion-mediated hopping events rather than with band-like conduction.^77^ In those systems, cation migration occurs within structurally organized coordination environments, supporting a transport scenario governed by ion-defined pathways instead of random diffusion. In the present framework, the κ^2^ and κ^3^ coordination pockets define such organized pathways, along which ionic migration and electron transfer become intrinsically coupled.

Notably, the ion–electron coupling mechanism identified here naturally leads to anisotropic charge-transport pathways, in line with experimentally reported redox-conduction behavior in this framework.^34^ More broadly, these results demonstrate that counterions play a nontrivial role in charge transport, acting as dynamic gates rather than passive charge compensators. This establishes a design principle whereby local ion–linker coordination that minimizes donor–acceptor reorganization and enhances electronic coupling between redox sites critically influences redox-hopping kinetics and charge-transport efficiency.

#### Ion-Mediated Charge Delocalization and Redox Conductivity

The emergence of the K^+^-bridged (pyrazole–NDI)_3_ trimer marks the culmination of the structural and electronic reorganization driving charge transport within the Zn­(pyrazole–NDI) framework. The stabilization of this tridentate configuration thus represents a structural signature of the ion-coupled redox-hopping regime in the Zn­(pyrazole–NDI) MOF.

This dynamic transfer regime is illustrated in Figure 8, which shows a representative AIMD snapshot (∼1300 fs) from a trajectory of an eight-unit-cell supercell, where sequential e^–^ injections (+1 e^–^ per unit cell every 500 fs) progressively drive the reduction of the framework. Panel (a) captures the formation of the K^+^-bridged (pyrazole–NDI)_3_ trimer, where a single K^+^ coordinates to three carbonyl oxygens (O_1_–O_3_) from neighboring linkers. Panels (b, c) contrast remote and local regions of the supercell: while the trimer-hosting layer exhibits a strong contraction of O···O distances, the remote layer (panel b) shows a substantial separation between pyrazole–NDI linkers, revealing that local K^+^ coordination simultaneously reorganizes the surrounding lattice and induces long-range structural polarization, thereby driving cooperative rearrangements and electrostatic coupling that propagate throughout the framework.

**Figure 8:**
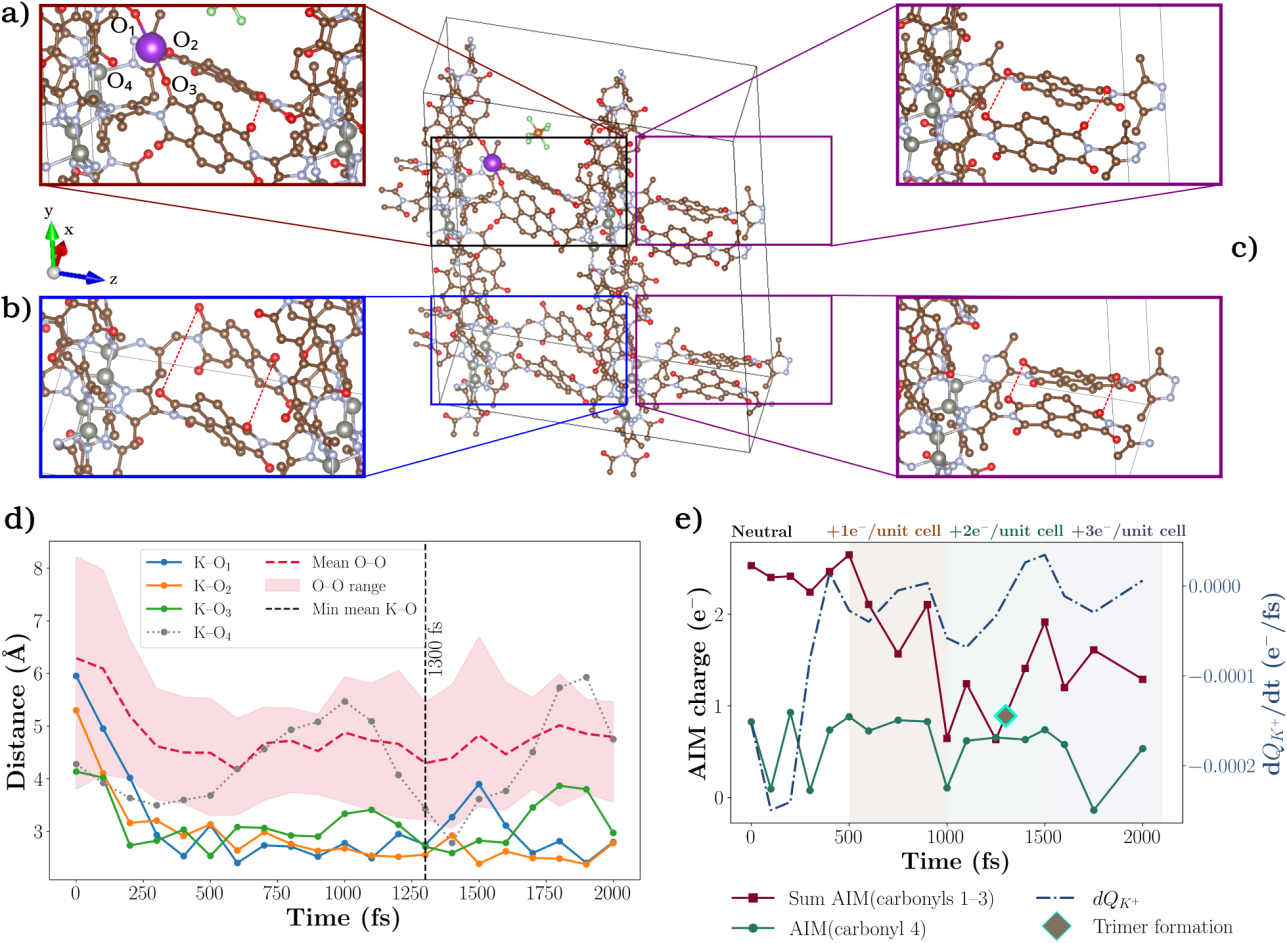
Counterion-mediated trimer formation and associated charge dynamics in Zn­(pyrazole–NDI). (a) Simulation snapshot at 1300 fs [Zn­(pyrazole–NDI•^–^) redox state], showing a K^+^ ion bridging three distinct carbonyl oxygens (O_1_, O_2_, O_3_) from adjacent pyrazole–NDI linkers, forming a transient c. (b) Representative region in a remote layer of the supercell, where equivalent carbonyl Os, unaffected by trimerization, remain widely separated (O···O: 7.34–8.37 Å). (c) Contrasting region where O···O distances (5.54–6.12 Å) remain short. (d) Time evolution of K–O (solid) and O–O (dashed red and shaded) distances among the three K^+^-coordinated carbonyls. The dashed vertical line at 1300 fs marks the global minimum in K–O distance, coinciding with maximal trimer stability; the gray dotted line tracks K–O_4_ (noncoordinating reference). (e) AIM charge dynamics: sum of charges for carbonyls 1–3 (dark red), reference carbonyl 4 (green), and dQ_K_^+^/dt (blue dashed). The diamond marks the moment of trimer formation (1300 fs), where a progressive depletion of the e^–^ density from the carbonyls is accompanied by increased K^+^ polarization. The subsequent accumulation of e^–^ density on the carbonyls and depolarization of K^+^ beyond 1500 fs highlight the transient nature of the trimeric assembly.

The temporal evolution of K–O and O–O distances (Figure 8d) quantifies the progression of trimer stabilization in the neutral state (anticipated from the PMF analysis). In the initial stages of the AIMD simulation, K^+^ is located away from the redox-active carbonyl centers of the NDI linkers. However, within the first 300 fs, even before any additional e^–^ are introduced, K^+^ rapidly migrates toward three specific carbonyl oxygens (O_1_, O_2_, O_3_). This migration is reflected in the sharp decrease of the K–O distances, accompanied by a notable contraction of the O–O distances between these K^+^-coordinated sites. This temporal contraction corroborates the structural reorganization inferred from the free-energy surfaces and marks the onset of full trimer stabilization during reduction. Importantly, the O–O distances plotted in Figure 8d correspond exclusively to carbonyl oxygens directly coordinated to K^+^, highlighting the emergence and sustained stability of this trimeric motif over time. By contrast, the distance to a fourth nearby carbonyl oxygen (O_4_, depicted as the gray dotted line) remains comparatively large for most of the simulation, indicating that K^+^ coordination is highly selective. Although the K^+^ distance to O_4_ briefly decreases at around 1400 fs, this contact is transient and does not persist.

As revealed by our combined PMF and CDFT analyses (Figure 6), the formation of trimeric K^+^-bridged [pyrazole–NDI] linker assemblies, arising from strong ion-pairing effects, enables through-bond charge transport, enhancing electronic connectivity across multiple crystallographic directions by reducing the spatial separation between adjacent redox-active centers. The contraction in interlinker redox center spatial separation is directly reflected by the sharp decline in the mean O–O distance, as illustrated by the red dashed curve in Figure 8d. This is not the first time that these effects have been detected through quantum mechanical calculations in MOFs with an interpenetrating structure.^62^

The emergence of a K^+^-coordinated trimeric [pyrazole–NDI] assembly (Figure 8a) is directly coupled to a pronounced structural response in a remote region of the supercell. Specifically, the NDI linkers situated in the adjacent lower layer (along the −*y* direction, with their π planes oriented approximately along *z*, Figure 8b) exhibit substantial spatial separation between their carbonyl groups. The maximum O–O distances between these carbonyl oxygens (Figure 8b) expand to 8.37 Å and 7.34 Å, markedly exceeding the much shorter O–O separations observed in other supercell regions (Figure 8c), where equivalent carbonyl pairs are only 5.54–6.12 Å apart. This contrast reveals that the formation of the K^+^–NDI trimer has a far-reaching impact on the supramolecular organization of the framework, acting as a structural switch that induces the cooperative rearrangement of distant linkers. This local motif triggers the dynamic opening of interlinker voids, potentially facilitating the transient transport of additional ionic or molecular species through the lattice.

A closer examination of the charge dynamics associated with trimer formation is provided in Figure 8e, which tracks the evolution of the sum of AIM charges for the three carbonyl groups directly coordinated to K^+^, the reference (uncoordinated) carbonyl, and the time derivative of the K^+^ AIM charge $\left(\frac{dQ_{K^{+}}}{dt}\right)$ . Notably, analyzing only the charges on the atoms of these carbonyl groups individually does not yield a clear indication of charge transfer processes. However, when the entire carbonyl units (C=O pairs) are considered, a well-defined electronic redistribution emerges, revealing that these carbonyl groups behave as integrated redox-active subsystems within each pyrazole–NDI linker. They transiently accumulate and release e^–^ density in response to fluctuations in the local coordination environment and to the progressive level of framework reduction, maintaining this dynamic exchange with K^+^ throughout the lifetime of the trimeric configuration.

Focusing on the 1250–1500 fs interval (Figure 8e, dark red curve), the combined AIM charge on the carbonyl groups becomes progressively less negative, indicating a gradual depletion of e^–^ density from these sites. At the same time, the AIM charge associated with K^+^ decreases smoothly, consistent with the positive slope in the dQ­(K^+^)/dt curve. This parallel evolution of charges demonstrates a direct and continuous electron transfer from the carbonyl units to K^+^, driven by the formation and stabilization of the K^+^ bridged trimer around ∼1300 fs. In contrast, the reference carbonyl group (C=O_4_, green curve), which does not participate in the trimer, shows no discernible trend. After ∼1500 fs, the reversed behavior in both quantities highlights the transient character of the trimeric assembly.

Importantly, our analysis reveals that this ion coordination also correlates with the emergence of valence symmetry breaking in the Zn­(pyrazole–NDI•^–0.5^) state (partially reduced or *x* = 0.5). As shown in Figure S4, the redox composition reshapes the local K^+^ coordination landscape: at *x* ≈ 0.5, a linker directly bound to K^+^ can accumulate more e^–^ density than a distant unbound linker, producing a charge imbalance consistent with mixed-valence behavior, whereas at higher reduction levels the charge distribution becomes progressively more uniform. These results demonstrate that K^+^ ions not only drive trimer formation and through-bond charge delocalization but also induce a controlled breaking of the framework’s pristine electronic symmetry. Importantly, hybrid-DFT validation (HSE06, 25% exact exchange) on the same maximum-stability trimer snapshot used in the AIMD/PMF analysis preserves the same symmetry-broken charge-localization pattern in the Zn­(pyrazole–NDI•^–0.5^) regime (Figure S5), indicating that the effect is not simply a PBE/GGA delocalization artifact. While a full hybrid-functional dynamical treatment of the extended framework remains beyond the present scope, the targeted PBE0/HSE06 calculations presented in the SI support the qualitative robustness of the central mechanistic picture with respect to the exchange-correlation treatment. By coupling local coordination dynamics with the global electronic structure, K^+^ acts as a dynamic gate for electron localization and delocalization, establishing a self-regulated redox-hopping equilibrium that governs the enhanced conductivity of the reduced Zn­(pyrazole–NDI) framework. Consistent with this interpretation, additional UV–vis calculations including explicit K^+^ show that the K^+^-bridged trimer reproduces the half-reduced experimental spectral profile substantially better than the corresponding model without K^+^, particularly in the absorption region above 400 nm (Figure S6).

Collectively, these results suggest that while the carbonyl groups accumulate e^–^ density under reduction conditions (e^–^-injections), they also act as transient e^–^-density contributors to nearby cations upon the formation of K^+^-bridged dimeric/trimeric assemblies. These assemblies not only mediate through-bond charge transfer, via orbital overlap between metal nodes and linker functional groups,^43^ but also reinforce electronic coupling between redox-active sites within and across linkers, facilitating e^–^-hopping and promoting redox conductivity throughout the framework. Consequently, optimizing the e^–^ transfer interactions between redox-active linker centers and strategically selected countercations becomes crucial, as this interaction supports the formation of trimeric or even higher-order assemblies that significantly boost electronic communication.

These simulations suggest that employing countercations capable of simultaneously coordinating multiple redox sites could amplify charge delocalization and enhance redox conductivity in MOF-based materials. In fact, redox conductivity measurements on thin films of Zn­(pyrazole–NDI) have demonstrated that the choice of ion significantly affects the conduction efficiency. Li^+^, for example, exhibits strong ion-pairing effects, whereas larger cations such as tetrabutylammonium (TBA^+^) influence electronic mobility through steric and solvation effects.^34^ The observed K^+^-bridged charge delocalization aligns with these experimental trends, reinforcing the view that certain counterions actively coparticipate in redox hopping by coordinating to redox-active ligands. Beyond a purely diffusive description of e^–^ hopping, our results reveal the active role of redox-inactive K^+^ counterions in modulating charge transport. Importantly, these trimeric arrangements should be viewed as transient, local coordination motifs whose population depends on the steric and solvation environment rather than as rigid structural units that must persist uniformly throughout the device. Moreover, defects, grain boundaries, and partial hydration in real thin films would be expected to modify the population, lifetime, and connectivity of these favorable local motifs. In particular, structural disorder may disrupt some locally accessible ion-bridged geometries, broaden the distribution of donor–acceptor separations and orientations, and thereby reduce the statistical prevalence of the lowest-barrier hopping channel. Partial hydration or local solvation can further compete with direct ion–linker coordination, altering the stability of the cooperative bidentate/tridentate states identified here. However, such a disorder does not invalidate the underlying mechanistic picture. Rather, it would be expected to modulate its quantitative expression by reducing the frequency and persistence of local ion-mediated configurations that promote charge delocalization. In this sense, disorder is more likely to attenuate the efficiency of the low-barrier pathway than to eliminate the ion-coupled redox-hopping mechanism itself.

The above interpretation, regarding the role of K^+^ ions in modulating transport, aligns with recent experimental evidence,^78^ where transient electrochemical measurements demonstrate that ion migration under internal electric fields directly contributes to the observed current response, and that ion pairing with reduced NDIs can give rise to an ion–electron coupled hopping mechanism. Our theoretical description is fully consistent with this scenario: the migration of K^+^ induced by the internal fields (generated in our simulations through successive reduction) dynamically couples to electronic motion, stabilizing transient states such as K^+^-coordinated dimeric/trimeric assemblies (Figures [Fig fig6]6a,b and 8). We observe that K^+^ can form ion pairs with reduced NDIs, leading to an ion–electron coupled hopping mechanism in which counterion polarization follows electronic charge redistribution. This coupling (i) promotes the formation of transient supramolecular assemblies that reinforce interlinker connectivity, (ii) enables directional transport within the framework, and (iii) stabilizes symmetry-broken configurations at half reduction (*x* = 0.5). Comparison with the intrinsic e^–^-hopping barriers computed in the absence of counterions (Δ*G* ^‡^_IEH_ = 120–154 meV) further clarifies this hierarchy: the K^+^-assisted bidentate and tridentate bridges (≈ 49.8 and 41.5 meV, respectively, for *x* = 0.5) define the cooperative, low-barrier limit of the redox-conducting regime, whereas Li^+^ and TBA^+^ exemplify the localized, high-barrier extremes where such ion-mediated delocalization is structurally impeded. Hence, the K^+^-bridged configuration emerges as the optimal balance between ionic size, coordination flexibility, and electronic coupling, serving as a structural archetype for counterion-gated charge transport in redox-active MOFs. More generally, the efficiency of this ion–electron coupled hopping mechanism is expected to depend on the size, coordination preferences, and bridging ability of the cation.

## Conclusions

This study establishes a comprehensive theoretical framework for charge transport in the Zn­(pyrazole–NDI) MOF, demonstrating that e^–^ conduction proceeds via redox hopping between spatially discrete pyrazole–NDI centers rather than band-like transport. Using AIMD simulations coupled with electronic-structure analysis, we reveal how e^–^ charge distribution, framework dynamics, and ion coordination collectively shape the redox conductive response.

Upon reduction, electron density first localizes on the imide N and carbonyl C/O atoms of the NDI core with only modest enrichment of the aromatic π-system; at higher reduction levels, the pyrazole N atoms are progressively engaged as secondary redox centers. This site-selective, stepwise occupation of frontier orbitals—initially lower-energy states with dominant carbonyl/imide character and subsequently higher-energy states with stronger pyrazole N character—defines the conduction pathway. The Zn^2+^ coordination environment enforces a rigid linker geometry that shapes this electronic landscape, with changes in NDI dihedrals and O–O distances modulating electronic communication between neighboring linkers. In the absence of counterions, the partially reduced state constitutes an intrinsic structural optimum for redox hopping: coordinated linker coplanarity, nodal-plane alignment, and transient O­(carbonyl)···H­(pyrazole) contacts collectively reinforce dynamic electron delocalization and short-range charge redistribution. By contrast, the singly reduced state accumulates geometric and electronic strain that disrupts these cooperative modes and diminishes the charge-carrier mobility.

Beyond this intrinsic response, our simulations uncover a strong coupling between ionic motion and electronic delocalization upon the introduction of K^+^. Reduction preorganizes carbonyl-rich linkers along the *z*-axis, facilitating K^+^ incorporation into inner-sphere coordination pockets and enabling the emergence of bidentate and tridentate motifs with markedly reduced intrinsic electron-hopping barriers. These K^+^-centered motifs exhibit cooperative ion–electron coupling, wherein the cation acts simultaneously as an electrostatic anchor and dynamic redox mediator, modulating electronic coupling as it migrates within its coordination environment.

K^+^ ions further promote the formation of transient dimeric and trimeric assemblies that reorganize redox connectivity and enhance through-space and through-bond interactions between NDI units. These assemblies highlight that counterions are not passive but actively reshape the charge-transport landscape via polarizing Coulombic interactions and local charge-density asymmetry within the partially reduced lattice. Ion coordination thus emerges as a tunable design axis for enabling anisotropic linker-mediated charge mobility. These mechanistic insights suggest that neuromorphic MOFs should be designed not simply for high redox conductivity but for dynamically reconfigurable ion–electron transport. In this context, redox-active linkers with accessible coordination sites, mixed-valence regimes, and cation environments that stabilize transient low-barrier hopping motifs may enable adaptive, spatially distributed, and history-dependent charge-transport responses analogous to those required for neuromorphic operation. Together, these findings provide a coherent microscopic description of ion-electron coupling in Zn­(pyrazole–NDI), establishing guiding principles for engineering MOF-based materials with enhanced redox performance for neuromorphic and electrochemical applications.

## Supplementary Material



## Notes

Computational Details; Structural and Electronic Characterization of Zn­(pyrazole–NDI) MOF via AIMD Simulations; Supporting Analysis of Structural Fluctuations via Filtered Oscillation Amplitude; AIM Charge Redistribution During Sequential Reductions; Extended Analysis of Redox-Induced Structural Reorganization; Details for Electron Transfer Coupling (Figure 5a); Hybrid-DFT validation of symmetry-broken charge localization in the partially reduced Zn­(pyrazole–NDI•^–0.5^) framework; Calculated UV–vis absorption spectra for the partially reduced Zn­(pyrazole–NDI) framework obtained without K^+^ and with explicit K^+^; Comparison of the PDOS obtained at the PBE and PBE0 (25% exact exchange) levels for the optimized unit cell of Zn­(pyrazole–NDI); Counterion Concentration and Trimer-Mediated Charge Dynamics; AIMD-Optimized Unit Cell Structure (PDF)

## References

[1] James S. L. (2003). Metal-organic
frameworks. Chem. Soc. Rev..

[2] Kitagawa S. (2014). Metal–organic
frameworks (MOFs). Chem. Soc. Rev..

[3] Elango I., Kumbhar D. D., Ambole Y. V., Abdullah M., Patil A. R., Selvamani M., Dongale T. D., Kim T. G., Kesavan A. V. (2025). Exploration
of Nonvolatile Memory and Synaptic Learning of a Metal–Organic
Framework (ZIF-67) and 2D Material (MoS2) Composite Memristor. ACS Appl. Electron. Mater..

[4] Li Z., Chen M.-H., Wu Q.-Q., Yuan C., Xu J.-J., Chen H.-Y., Zhao W.-W. (2025). A metal-organic framework neuron. Nat. Sci. Rev..

[5] Shaharukh S., Sadhukhan R., Sangwan S. K., Goswami D. K., Dhar A. (2025). A MOF-embedded
multifunctional dielectric layer for an energy-efficient organic memtransistor
and brain-inspired computing. J. Mater. Chem.
C.

[6] Zhu D., Du J., Peng Z., Wang J., He X., Li G., Ye L., Ling H., Zhao M., Lin H. (2025). Metal–Organic
Frameworks Coordination-Oriented Polymer Dielectrics for Neuromorphic
Vision Sensors. SmartMat.

[7] Bhosale S. V., Jani C. H., Langford S. J. (2008). Chemistry of naphthalene diimides. Chem. Soc. Rev..

[8] Andric G., Boas J. F., Bond A. M., Fallon G. D., Ghiggino K. P., Hogan C. F., Hutchison J. A., Lee M. A.-P., Langford S. J., Pilbrow J. R. (2004). Spectroscopy
of naphthalene diimides and their
anion radicals. Aust. J. Chem..

[9] Al
Kobaisi M., Bhosale S. V., Latham K., Raynor A. M., Bhosale S. V. (2016). Functional naphthalene diimides: synthesis, properties,
and applications. Chem. Rev..

[10] Katz H., Lovinger A., Johnson J., Kloc C., Siegrist T., Li W., Lin Y.-Y., Dodabalapur A. (2000). A soluble and air-stable organic
semiconductor with high electron mobility. Nature.

[11] Vasimalla S., Senanayak S. P., Sharma M., Narayan K., Iyer P. K. (2014). Improved
performance of solution-processed n-type organic field-effect transistors
by regulating the intermolecular interactions and crystalline domains
on macroscopic scale. Chem. Mater..

[12] Ko M., Lee Y., Jo Y., Jang J. H., Lee M. J. (2019). Analysis of enhanced
hole transport in naphthalene dicarboxyimide (NDI)-based n-type polymer
field-effect transistors using solution-processed reduced graphene
oxide electrodes. Appl. Surf. Sci..

[13] Jain S., Sridevi M., Majhi T., Tripathi N. P., Sengupta S., Singh R. K. (2024). Unraveling the Optoelectronic
Exciton Dynamics of NDI
(Naphthalene Diimide)-Based Nonfullerene Acceptor with Improved Band
Alignment. ACS Appl. Polym. Mater..

[14] Sung M. J., Huang M., Moon S. H., Lee T. H., Park S. Y., Kim J. Y., Kwon S.-K., Choi H., Kim Y.-H. (2017). Naphthalene
diimide-based small molecule acceptors for fullerene-free organic
solar cells. Solar Energy.

[15] Liu Y., Page Z. A., Russell T. P., Emrick T. (2015). Finely tuned polymer
interlayers enhance solar cell efficiency. Angew.
Chem., Int. Ed..

[16] Rest C., Kandanelli R., Fernández G. (2015). Strategies to create hierarchical
self-assembled structures via cooperative non-covalent interactions. Chem. Soc. Rev..

[17] Babu S. S., Praveen V. K., Ajayaghosh A. (2014). Functional
π-gelators and their
applications. Chem. Rev..

[18] Guha S., Goodson F. S., Corson L. J., Saha S. (2012). Boundaries of anion/naphthalenediimide
interactions: from anion– π interactions to anion-induced
charge-transfer and electron-transfer phenomena. J. Am. Chem. Soc..

[19] Das A., Ghosh S. (2016). H-bonding directed
programmed supramolecular assembly of naphthalene-diimide
(NDI) derivatives. Chem. Commun..

[20] Praveen V.
K., Ranjith C., Armaroli N. (2014). White-light-emitting supramolecular
gels. Angew. Chem., Int. Ed..

[21] Babu S. S., Prasanthkumar S., Ajayaghosh A. (2012). Self-assembled
gelators for organic
electronics. Angew. Chem., Int. Ed..

[22] Seki S., Saeki A., Sakurai T., Sakamaki D. (2014). Charge carrier mobility
in organic molecular materials probed by electromagnetic waves. Phys. Chem. Chem. Phys..

[23] Schenning A. P., Meijer E. (2005). Supramolecular electronics;
nanowires from self-assembled
π-conjugated systems. Chem. Commun..

[24] Kim S. H., Parquette J. R. (2012). A model
for the controlled assembly of semiconductor
peptides. Nanoscale.

[25] Aida T., Meijer E., Stupp S. (2012). Functional
supramolecular polymers. Science.

[26] Maeda H. (2009). Acyclic oligopyrroles
as building blocks of supramolecular assemblies. J. Inclusion Phenom. Macrocyclic Chem..

[27] Li W.-S., Saeki A., Yamamoto Y., Fukushima T., Seki S., Ishii N., Kato K., Takata M., Aida T. (2010). Use of Side-Chain Incompatibility
for Tailoring Long-Range p/n Heterojunctions:
Photoconductive Nanofibers Formed by Self-Assembly of an Amphiphilic
Donor–Acceptor Dyad Consisting of Oligothiophene and Perylenediimide. Chem. - Asian J..

[28] Honsho Y., Miyakai T., Sakurai T., Saeki A., Seki S. (2013). Evaluation
of intrinsic charge carrier transport at insulator-semiconductor interfaces
probed by a non-contact microwave-based technique. Sci. Rep..

[29] Saeki A., Ohsaki S.-I., Seki S., Tagawa S. (2008). Electrodeless determination
of charge carrier mobility in poly (3-hexylthiophene) films incorporating
perylenediimide as photoconductivity sensitizer and spectroscopic
probe. J. Phys. Chem. C.

[30] Saeki A., Seki S., Koizumi Y., Tagawa S. (2007). Dynamics of photogenerated
charge carrier and morphology dependence in polythiophene films studied
by in situ time-resolved microwave conductivity and transient absorption
spectroscopy. J. Photochem. Photobiol., A.

[31] Yagai S., Usui M., Seki T., Murayama H., Kikkawa Y., Uemura S., Karatsu T., Kitamura A., Asano A., Seki S. (2012). Supramolecularly engineered
perylene bisimide assemblies exhibiting
thermal transition from columnar to multilamellar structures. J. Am. Chem. Soc..

[32] Adhikari B., Lin X., Yamauchi M., Ouchi H., Aratsu K., Yagai S. (2017). Hydrogen-bonded
rosettes comprising π-conjugated systems as building blocks
for functional one-dimensional assemblies. Chem.
Commun..

[33] Takahashi S., Matsumoto T., Hollamby M. J., Miyasaka H., Vacha M., Sotome H., Yagai S. (2024). Impact of Ring-Closing on the Photophysical
Properties of One-Dimensional π-Conjugated Molecular Aggregate. J. Am. Chem. Soc..

[34] Li J., Kumar A., Johnson B. A., Ott S. (2023). Experimental manifestation
of redox-conductivity in metal-organic frameworks and its implication
for semiconductor/insulator switching. Nat.
Commun..

[35] Takenaka S. (2021). Application
of naphthalene diimide in biotechnology. Polym.
J..

[36] Hendon C. H., Rieth A. J., Korzynski M. D., Dinca M. (2017). Grand challenges and
future opportunities for metal–organic frameworks. ACS Cent. Sci..

[37] Duan J., Goswami S., Patwardhan S., Hupp J. T. (2022). Does the Mode of
Metal–Organic Framework/Electrode Adhesion Determine Rates
for Redox-Hopping-Based Charge Transport within Thin-Film Metal–Organic
Frameworks?. J. Phys. Chem. C.

[38] Qian Q., Asinger P. A., Lee M. J., Han G., Mizrahi
Rodriguez K., Lin S., Benedetti F. M., Wu A. X., Chi W. S., Smith Z. P. (2020). MOF-based membranes
for gas separations. Chem. Rev..

[39] Li Y., Yang R. T. (2007). Gas adsorption
and storage in metal– organic
framework MOF-177. Langmuir.

[40] Wang Q., Astruc D. (2020). State of the art and
prospects in metal–organic
framework (MOF)-based and MOF-derived nanocatalysis. Chem. Rev..

[41] García-García P., Müller M., Corma A. (2014). MOF catalysis in relation to their
homogeneous counterparts and conventional solid catalysts. Chem. Sci..

[42] Ren J., Huang Y., Zhu H., Zhang B., Zhu H., Shen S., Tan G., Wu F., He H., Lan S. (2020). Recent progress on MOF-derived
carbon materials for
energy storage. Carbon Energy.

[43] Talin A. A., Centrone A., Ford A. C., Foster M. E., Stavila V., Haney P., Kinney R. A., Szalai V., El Gabaly F., Yoon H. P. (2014). Tunable
electrical conductivity in metal-organic framework
thin-film devices. Science.

[44] Dong R., Han P., Arora H., Ballabio M., Karakus M., Zhang Z., Shekhar C., Adler P., Petkov P. S., Erbe A. (2018). High-mobility band-like
charge transport in a semiconducting two-dimensional
metal–organic framework. Nat. Mater..

[45] Chuang C.-H., Li J.-H., Chen Y.-C., Wang Y.-S., Kung C.-W. (2020). Redox-hopping
and electrochemical behaviors of metal–organic framework thin
films fabricated by various approaches. J. Phys.
Chem. C.

[46] Nyakuchena J., Ostresh S., Streater D., Pattengale B., Neu J., Fiankor C., Hu W., Kinigstein E. D., Zhang J., Zhang X. (2020). Direct evidence of photoinduced charge
transport mechanism in 2D conductive metal organic frameworks. J. Am. Chem. Soc..

[47] Cai M., Loague Q., Morris A. J. (2020). Design rules for efficient charge
transfer in metal–organic framework films: the pore size effect. J. Phys. Chem. Lett..

[48] Johnson E. M., Ilic S., Morris A. J. (2021). Design
strategies for enhanced conductivity
in metal–organic frameworks. ACS Cent.
Sci..

[49] Parashar R. K., Jash P., Zharnikov M., Mondal P. C. (2024). Metal-organic Frameworks
in Semiconductor Devices. Angew. Chem., Int.
Ed..

[50] Goswami S., Hod I., Duan J. D., Kung C.-W., Rimoldi M., Malliakas C. D., Palmer R. H., Farha O. K., Hupp J. T. (2019). Anisotropic redox
conductivity within a metal–organic framework material. J. Am. Chem. Soc..

[51] Wang Z., Nminibapiel D., Shrestha P., Liu J., Guo W., Weidler P. G., Baumgart H., Wöll C., Redel E. (2016). Resistive switching
nanodevices based on metal–organic frameworks. ChemNanoMat.

[52] Zhao L., Wu W., Shen X., Liu Q., He Y., Song K., Li H., Chen Z. (2019). Nonvolatile electrical bistability behaviors observed
in Au/Ag nanoparticle-embedded MOFs and switching mechanisms. ACS Appl. Mater. Interfaces.

[53] Kulachenkov N., Haar Q., Shipilovskikh S., Yankin A., Pierson J.-F., Nominé A., Milichko V. A. (2022). MOF-Based Sustainable Memory Devices. Adv. Funct. Mater..

[54] Liu Y., Fischer F., Hu H., Gliemann H., Natzeck C., Schwotzer M., Rainer C., Lemmer U., Wöll C., Breitung B. (2025). Inkjet Printed Metal–Organic Frameworks
for Non-Volatile Memory Devices Suitable for Printed RRAM. Adv. Funct. Mater..

[55] Cai G., Liu Z., Yang J., Xie H., Yu X., Zheng B. (2024). The synthesis
of MOF nanosheets and their application in MOF-based resistance random
access memory devices. J. Mater. Chem. C.

[56] Zhang X., Da Silva I., Fazzi R., Sheveleva A. M., Han X., Spencer B. F., Sapchenko S. A., Tuna F., McInnes E. J. L., Li M. (2019). Iodine
adsorption in a redox-active metal–organic
framework: Electrical conductivity induced by Host– Guest charge-transfer. Inorg. Chem..

[57] Hod I., Farha O. K., Hupp J. T. (2016). Modulating the rate of charge transport
in a metal–organic framework thin film using host: guest chemistry. Chem. Commun..

[58] Hod I., Bury W., Gardner D. M., Deria P., Roznyatovskiy V., Wasielewski M. R., Farha O. K., Hupp J. T. (2015). Bias-switchable
permselectivity and redox catalytic activity of a ferrocene-functionalized,
thin-film metal–organic framework compound. J. Phys. Chem. Lett..

[59] Gracia R., Mecerreyes D. (2013). Polymers with
redox properties: materials for batteries,
biosensors and more. Polym. Chem..

[60] Karlsson C., Suga T., Nishide H. (2017). Quantifying
TEMPO redox polymer charge
transport toward the organic radical battery. ACS Appl. Mater. Interfaces.

[61] Mohammad-Pour G. S., Hatfield K. O., Fairchild D. C., Hernandez-Burgos K., Rodríguez-López J., Uribe-Romo F. J. (2019). A solid-solution
approach for redox active metal–organic frameworks with tunable
redox conductivity. J. Am. Chem. Soc..

[62] Castner A. T., Su H., Svensson Grape E., Inge A. K., Johnson B. A., Ahlquist M. S., Ott S. (2022). Microscopic insights into cation-coupled
electron hopping transport in a metal–organic framework. J. Am. Chem. Soc..

[63] Qu L., Iguchi H., Takaishi S., Habib F., Leong C. F., D’Alessandro D. M., Yoshida T., Abe H., Nishibori E., Yamashita M. (2019). Porous molecular conductor: electrochemical
fabrication of through-space conduction pathways among linear coordination
polymers. J. Am. Chem. Soc..

[64] Paulsen B. D., Tybrandt K., Stavrinidou E., Rivnay J. (2020). Organic mixed ionic–electronic
conductors. Nat. Mater..

[65] Berkbigler G., Liu Q., Hoefer N., Xie Y., Hilliard J. S., McComb D. W., Wade C. R. (2024). C2H2/CO2 Separation
with a Chain-Type Zn Pyrazolate
MOF. Eur. J. Inorg. Chem..

[66] Thurston J. R., Li S., Sun Q., Nordlund D., Iglesias L. K., Sindt C., Kumar S., Grinter D. C., Li H., Greenaway A. L. (2025). Spectroscopic Investigation into P (NDI2OD-T2)
Charge Localization. Chem. Mater..

[67] Crameri F., Shephard G. E., Heron P. J. (2020). The misuse
of colour in science communication. Nat. Commun..

[68] Castro-Castillo C., Suazo-Hernández J., Espinoza-González R., Garcia G. (2025). Smart Materials for
Carbon Neutrality: Redox-Active
MOFs for Atmospheric CO2 Capture by Electrochemical Methods. Catalysts.

[69] Rath B. B., Lotsch B. V. (2026). Ion Diffusion and
(Photo) redox Conductivity in a Covalent
Organic Framework. J. Am. Chem. Soc..

[70] Shavaleev N. M., Davies E. S., Adams H., Best J., Weinstein J. A. (2008). Platinum
(II) diimine complexes with catecholate ligands bearing imide electron-acceptor
groups: Synthesis, crystal structures,(spectro) electrochemical and
EPR studies, and electronic structure. Inorg.
Chem..

[71] Zeraati M., Alizadeh V., Kazemzadeh P., Safinejad M., Kazemian H., Sargazi G. (2022). A new nickel metal
organic framework
(Ni-MOF) porous nanostructure as a potential novel electrochemical
sensor for detecting glucose. J. Porous Mater..

[72] Gibbons B., Cairnie D. R., Thomas B., Yang X., Ilic S., Morris A. J. (2023). Photoelectrochemical water oxidation by a MOF/semiconductor
composite. Chem. Sci..

[73] Weng Y.-G., Ren Z.-H., Zhang Z.-R., Shao J., Zhu Q.-Y., Dai J. (2021). Tetrathiafulvalene–cobalt
metal–organic frameworks
for lithium-ion batteries with superb rate capability. Inorg. Chem..

[74] Xie L. S., Skorupskii G., Dinca M. (2020). Electrically conductive metal–organic
frameworks. Chem. Rev..

[75] Seyffertitz M., Balhatchet C. J., Rauscher M. V., Stock S., Fritz-Popovski G., Leiner T., Holec D., Amenitsch H., Forse A. C., Paris O. (2025). Selective anion anchoring in MOF-based
supercapacitors revealed with operando small-angle X-ray scattering. Nat. Commun..

[76] Daliran S., Oveisi A. R., Peng Y., López-Magano A., Khajeh M., Mas-Ballesté R., Alemán J., Luque R., Garcia H. (2022). Metal–organic
framework (MOF)-,
covalent-organic framework (COF)-, and porous-organic polymers (POP)-catalyzed
selective C–H bond activation and functionalization reactions. Chem. Soc. Rev..

[77] Tao S., Liu R., Mu X., Ye X., Niu X., Yang S.-W., Jiang D. (2026). Potassium-Ion Conduction
in Covalent Organic Frameworks. J. Am. Chem.
Soc..

[78] Johnson B. A., Castner A. T., Agarwala H., Ott S. (2025). Beyond diffusion: ion
and electron migration contribute to charge transport in redox-conducting
metal–organic frameworks. Chem. Sci..

