# Supplementary material for: Ion-Electron Coupling-Driven Redox Behavior in Metal–Organic Frameworks
Source: J Am Chem Soc. 2026 Apr 21;148(17):18346–64. doi: 10.1021/jacs.6c03704 (PMC13154206; doi:10.1021/jacs.6c03704)
Supplement: Supplementary file 1 [file ja6c03704_si_001.pdf]

Supplementary Information

Ion-Electron Coupling-Driven Redox Behavior in Metal–Organic Frameworks

A. Avilés,[1]* M. Ghotbi,[2] A. J. Ferguson,[4] J. L. Blackburn,[4] A. A. Talin,[5] M. Y. Darensbourg,[2] P. B. Balbuena[1,2,3]*

[1]Department of Chemical Engineering, Texas A&M University, College Station, TX 77843; USA
[2]Department of Chemistry, Texas A&M University, College Station, TX 77843; USA
[3]Department of Materials Science and Engineering, Texas A&M University, College Station, TX 77843, USA
[4]Chemistry and Nanoscience Center, National Laboratory of the Rockies (NLR), Golden, CO 80401, USA
[5] Chemistry, Combustion and Materials Science Department, Sandia National Laboratories, Livermore, CA, 94550, USA

*e-mails: aaviles@tamu.edu; balbuena@tamu.edu

Computational Details

The structure of the Zn(pyrazole-NDI) MOF was reconstructed based on reference 34 and subsequently optimized using DFT within the projector augmented wave (PAW) formalism as implemented in the VASP software. [1] The optimization was performed *via* conjugate gradient minimization, utilizing a Γ-centered k-point mesh of 4 × 4 × 4 and a plane-wave energy cutoff of 400.0 eV. Exchange-correlation effects were treated using the Perdew–Burke–Ernzerhof (PBE) [2] functional within the framework of the generalized gradient approximation (GGA).

AIMD simulations [1-3] were performed to investigate the dynamic interactions between electronic and structural changes during the successive reduction processes in the Zn(pyrazole-NDI) MOF. These simulations were conducted under finite temperature conditions using a Nosé-Hoover thermostat [4] to ensure thermal control. The AIMD approach was chosen to capture dynamic effects that influence charge transport mechanisms, allowing for a more accurate representation of the system's response to reduction. A timestep of 1.0 fs was used to accurately resolve atomic motion and electronic interactions throughout the simulation. To simulate the experimentally observed redox processes in MOF thin films, [5] extra e^- were sequentially introduced into the unit cell containing two redox centers, to model the stepwise reduction: i) 2 Zn(pyrazole-NDI) + $1e^-$ → 2Zn(pyrazole-NDI$\bullet^{-0.5}$), and ii) 2Zn(pyrazole-NDI$\bullet^{-0.5}$) + $1e^-$ → 2Zn(pyrazole-NDI$\bullet^{-}$). This methodology enabled a detailed evaluation of the energetic and structural stability of the system across the neutral, partially-reduced, and singly-reduced states, while also facilitating a precise exploration of the electronic and structural changes in the Zn(pyrazole-NDI) MOF throughout the reduction processes.

Additionally, an atomic charge analysis was performed using the Bader's Atoms in Molecules (AIM) partitioning method, [6, 7] implemented through Henkelman's algorithm for charge density decomposition. [8] This analysis was conducted on selected atomic distributions, enabling a detailed evaluation of the electronic redistribution induced by the reduction processes in the Zn(pyrazole-NDI) MOF. The detailed analysis and visualization of atomic trajectories, AIM charge color maps, and

charge density isosurfaces were carried out using the OVITO software, [9] leveraging its Python interface for data post-processing.

To further investigate charge transport in the Zn(pyrazole-NDI) MOF architecture, we analyzed the temporal evolution of the charge carrier localization region ($\rho_e = f(t)$) during an AIMD simulation of a 2×2×2 supercell. The simulation was performed under a temperature ramp of 40K/ps to elevate the temperature from 200 K to 400 K with an additional e^- (+1e^-), allowing the system to progressively adapt to thermal and structural changes before analyzing charge localization dynamics. The e^- density associated with the charge carrier was identified by visualizing the charge difference isosurface at a fixed isovalue, derived from the difference in e^- density between the neutral reference and the reduced supercell. This procedure was applied to successive snapshots within the +1e^-/supercell reduction regime, enabling a direct, time-resolved comparison of charge localization. This approach allowed us to track the charge-hopping dynamics of the MOF's electronic structure, providing insights into its response to thermal and electronic perturbations.

To evaluate the coupled ion-electron transport mechanisms in the Zn(pyrazole–NDI) framework, we combined ab initio molecular dynamics (AIMD)–derived potential of mean force (PMF) analyses with constrained DFT (CDFT) calculations that allow evaluation of parameters needed to implement Marcus theory analysis. For the PMF[10]) calculations (Figure 6a,b), AIMD trajectories of both neutral and partially reduced lattices containing a single K[PF_6] unit were analyzed. We used a 2×1×1 minimal supercell containing two linkers in the lateral direction (x), which enables lateral coordination rearrangements and the formation of realistic K^+-bridged dimeric and trimeric motifs. The trajectories were projected onto two collective variables: (i) the smooth coordination number (CN) between K^+ and the O + N donor atoms of the linkers, and (ii) the third-shortest K–O distance (d_3), which captures the transition between bidentate (κ^2-O,O′) and tridentate (κ^3-O,O′,O″) coordination states. The PMF,

$$G(\xi) = -k_B T \, lnP(\xi) + C, \quad \text{(S1)}$$

was reconstructed from the normalized probability distribution $P(\xi)$ of these variables, yielding the free-energy barriers $\Delta G(\xi)$ associated with K^+ migration and local reorganization. In this equation, k_B is the Boltzmann constant, T is the absolute temperature, and C is an additive constant that defines the zero of free energy. Here, the AIMD trajectory was performed under a Langevin thermostat[11] in the canonical (NVT) ensemble; therefore, the probability distribution P(ξ) is already an ensemble-averaged quantity sampled according to $e^{-\beta U}$, where $\beta = 1/(k_B T)$ and U is the potential energy of the system. The resulting PMF represents a free-energy surface directly derived from unbiased canonical sampling. Complementary Marcus–CDFT calculations[12, 13] (Figure 7a,c) were performed in NWChem 7.2.3[14] using the hybrid PBE0 functional and open-shell configurations on an extracted donor–acceptor (D-A) cluster containing two pyrazole-NDI linkers and their Zn nodes (H-capped). Preliminary optimizations employed the 6-31G* + def2-SV(P) basis sets for light and heavy atoms, followed by refined single-point calculations with 6-311+G(2d,p) for accurate charge localization. Charge and spin localization constraints were applied to enforce diabatic states on the NDI fragments. From the resulting single-point energies E at fixed nuclear geometries Q for donor D and acceptor A – $E_D(Q_D)$, $E_A(Q_A)$, $E_D(Q_A)$, $E_A(Q_D)$ – the internal reorganization energy (λ_i), Gibbs free-energy difference (ΔG^0), and activation energy ($\Delta G^\ddagger$) were computed following the Marcus formalism[15, 16]. Within the Marcus framework, the internal reorganization energy was obtained as

$$\lambda_{in} = \frac{\lambda_D + \lambda_A}{2} = \frac{[E_D(Q_A) - E_D(Q_D)] + [E_A(Q_D) - E_A(Q_A)]}{2}, \quad \text{(S2)}$$

where $E_D(Q_D)$and $E_A(Q_A)$correspond to the donor- and acceptor-localized electronic states evaluated at their respective equilibrium geometries, and $E_D(Q_A)$and $E_A(Q_D)$ are the corresponding vertical energies at the opposite nuclear configurations. The intrinsic electron-hopping activation barrier was then evaluated as

$$\Delta G^{\ddagger}_{IEH} = \frac{(\Delta G^0)^2}{4\lambda_{in}}, \quad \text{(S3)}$$

where $\Delta G^0$is the reaction free-energy difference between the diabatic states. The combination of AIMD-PMF and CDFT-Marcus approaches provided a quantitative description of both ionic and electronic barriers, revealing their convergence into a shared, low-barrier adiabatic regime ($\Delta G^{\ddagger} \approx$ 45 meV) that defines the cooperative ion-electron hopping mechanism. Interaction Region Indicator (IRI) analyses [17] (Figure 7b,d) were subsequently performed on representative AIMD-derived clusters used for the CDFT calculations, employing Multiwfn 3.8[18] to compute the scalar fields and UCSF Chimera[19] for visualization of the resulting isosurfaces. This approach enabled identification and characterization of noncovalent interlinker interactions arising during K^+ coordination and reduction.

To elucidate the dynamic charge redistribution within the K^+-bridged (pyrazole–NDI)$_3$ trimer and its impact on redox conductivity, AIMD simulations were performed at 300 K on a 2×2×2 supercell of Zn(pyrazole-NDI) MOF (Figure 8). In all counterion-containing AIMD simulations, a single K[PF_6] unit was incorporated into the neutral supercell prior to electron injection and subsequently retained unchanged across all reduction steps. This strategy intentionally imposes a locally high counterion concentration and was designed to isolate the structural and electronic effects of K^+ coordination without introducing additional complexity from variable counterion stoichiometries. The protocol comprised an initial 500 fs equilibration in the neutral state, followed by three successive reduction steps: after each 500 fs interval, 8 additional e^- were incrementally introduced into the supercell, corresponding to +1, +2, and +3 extra e^- per formula unit, respectively. Although the simulated salt concentration is much higher than the experimental one, the simulation may represent a high local concentration environment. The temporal evolution of key K–O and O–O distances was systematically tracked throughout the simulation, enabling a direct correlation between counterion dynamics and the emergence of characteristic structural motifs at each redox state. All trajectory processing and charge localization analyses were conducted using custom Python scripts and VESTA.[20]

Structural and Electronic Characterization of Zn(pyrazole-NDI) MOF via AIMD Simulations

Based on 5000 fs AIMD simulations at T = 300 K, for the neutral system (blue graph, Figure S-1), the internal energy relative to its initial value displays a clear asymptotic trend after an initial equilibration period of 1000 fs, reaching an average value of approximately 3.16 eV. Although relatively large energy fluctuations (~±1.20 eV) are observed toward the end of the simulation and the timescale remains limited, no significant structural changes occur within the simulated window. Despite these oscillations, first principles simulations point to dynamic stability of the neutral system within the accessible timescale. The structural modeling of the Zn(pyrazole-NDI) MOF is validated by the excellent agreement between the simulated powder diffraction pattern and experimental X-ray diffraction (XRD) data for a thin film of Zn(pyrazole-NDI) on fluorine-doped tin oxide (FTO) surfaces[5] (Figure S-2). Notably, the top peaks in the simulated pattern appear at 2θ = 5.22°, 10.45°, and 12.14°, which closely match the most intense features observed experimentally. This

consistency confirms that the simulated unit cell structure after 5000 fs of AIMD accurately reproduces the experimental structural characteristics of the material in a thin film configuration. For reference and reproducibility, the final AIMD-optimized structure has been provided in the Supporting Information. To further verify the electronic properties, the final atomic configuration from the neutral-state AIMD simulation was used to compute the UV-Vis spectrum (Figure S-3). The neutral system exhibits absorption maxima at 370.51 nm and 343.70 nm, aligning well with the experimental π–π* transitions of the NDI core at 379 nm and 360 nm. [5]

For the partially-reduced state (Figure S-1), the relative internal energy stabilizes after 1000 fs, with a slightly higher average value of approximately 3.32 eV and a broader oscillation range of ~±1.31 eV. The addition of one e^- induces a redistribution of charge toward the NDI ligands, which serve as redox centers. This change introduces moderate energetic fluctuations, although the system retains overall structural stability. In the singly-reduced state (Figure S-1), the relative internal energy converges to an average value of approximately 3.40 eV, with oscillations comparable in amplitude (~±1.32 eV) to those observed in the partially-reduced state. The introduction of a second e^- does not significantly destabilize the system, indicating an efficient reorganization of electronic density in the NDI ligands to accommodate the additional charge. For the further reduced states (+3e^- and +4e^-, Figure S-1), the system exhibits distinct energetic trends. In both states, the relative free energy stabilizes at 3.59 eV, but their dynamic behaviors differ significantly. The +3e^- state maintains regular oscillations with a moderate amplitude (1.26 eV), indicating efficient charge delocalization and structural adaptability. In contrast, the +4e^- state shows greater fluctuations (1.36 eV) and irregular oscillations, suggesting charge localization or structural distortions. The fact that the asymptotic energy remains unchanged from the +3e^- to +4e^- state despite the increased oscillation amplitude reveals that, while the system can formally accept a fourth electron, the redistribution of charge becomes inefficient. These results demonstrate that while successive reductions lead to slight increases in the average relative free energy and oscillation amplitudes, the MOF maintains its energetic and structural stability across the three redox states. This behavior highlights the critical role of NDI ligands as robust redox centers, capable of supporting charge accumulation and facilitating electronic transport via the redox-hopping mechanism.

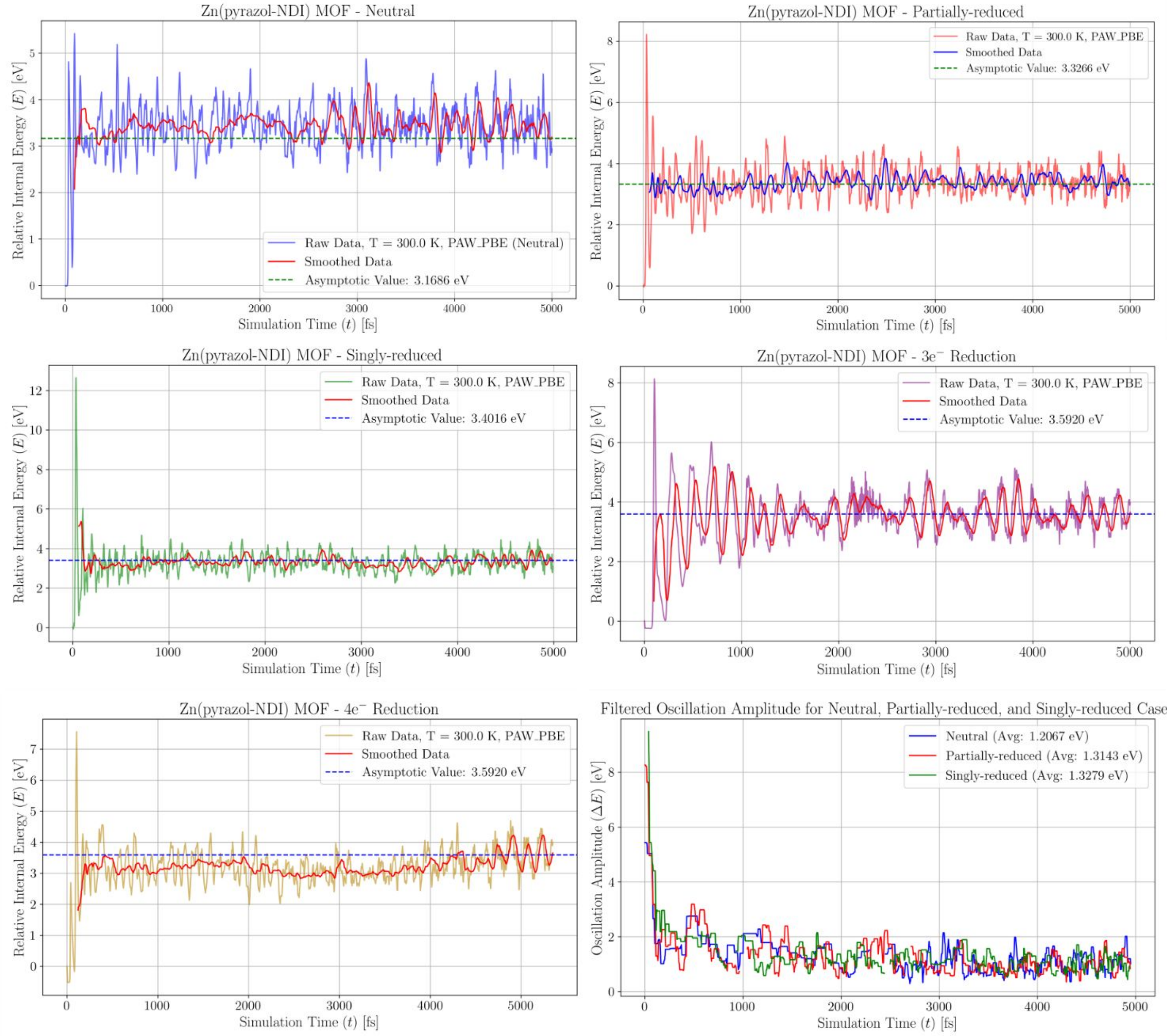

***Figure S-**1. Evolution of the relative internal energy and filtered oscillation amplitude in the Zn(pyrazole-NDI) MOF system under different redox states. Each panel shows the AIMD trajectory over 5000 fs at 300.0 K, comparing the raw internal energy data (blue or other colors), smoothed data (red), and the computed asymptotic value (dashed line). Relative energies are referenced to the initial energy at t = 0.0 fs. The key charge states shown are: neutral, partially-reduced, singly-reduced, and additionally +3e⁻ and +4e⁻ cases for comparison.*

Supporting Analysis of Structural Fluctuations via Filtered Oscillation Amplitude

The filtered oscillation amplitude (ΔE) was computed (lower right panel, Figure S-1) to quantify the extent of dynamic fluctuations around the stabilized configurations sampled during AIMD. This metric is defined as the instantaneous deviation between the raw internal energy and its smoothed counterpart, obtained via a moving average filter.

While all redox states exhibit energetic stabilization following the initial thermal relaxation, the neutral state maintains the lowest average ΔE throughout the simulation, indicating relatively constrained atomic motion. In contrast, the partially-reduced state presents slightly larger fluctuations, which may reflect increased structural flexibility or transient distortions induced by additional electronic charge.

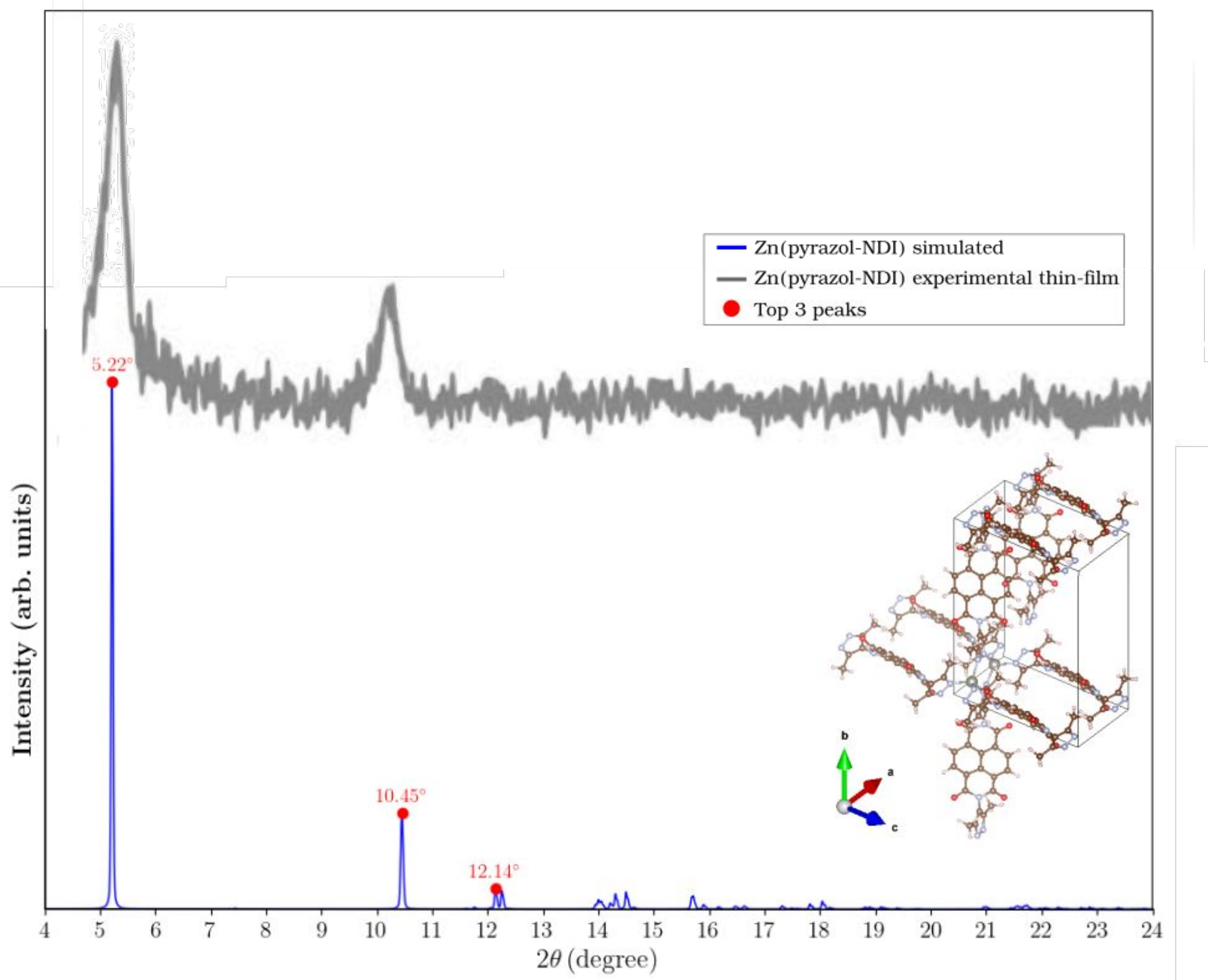

Figure S-2. *Simulated powder diffraction pattern of the MOF unit cell structure after 5000 fs of ab initio molecular dynamics, alongside experimental X-ray diffraction (XRD) data[5] for a thin film of Zn(pyrazole–NDI) on fluorine-doped tin oxide (FTO) surfaces. Experimental data adapted from [5]. Available under a CC-BY [4.0] International License. Copyright [2023] [Li, J.; Kumar, A.; Johnson, B. A.; Ott, S.]*

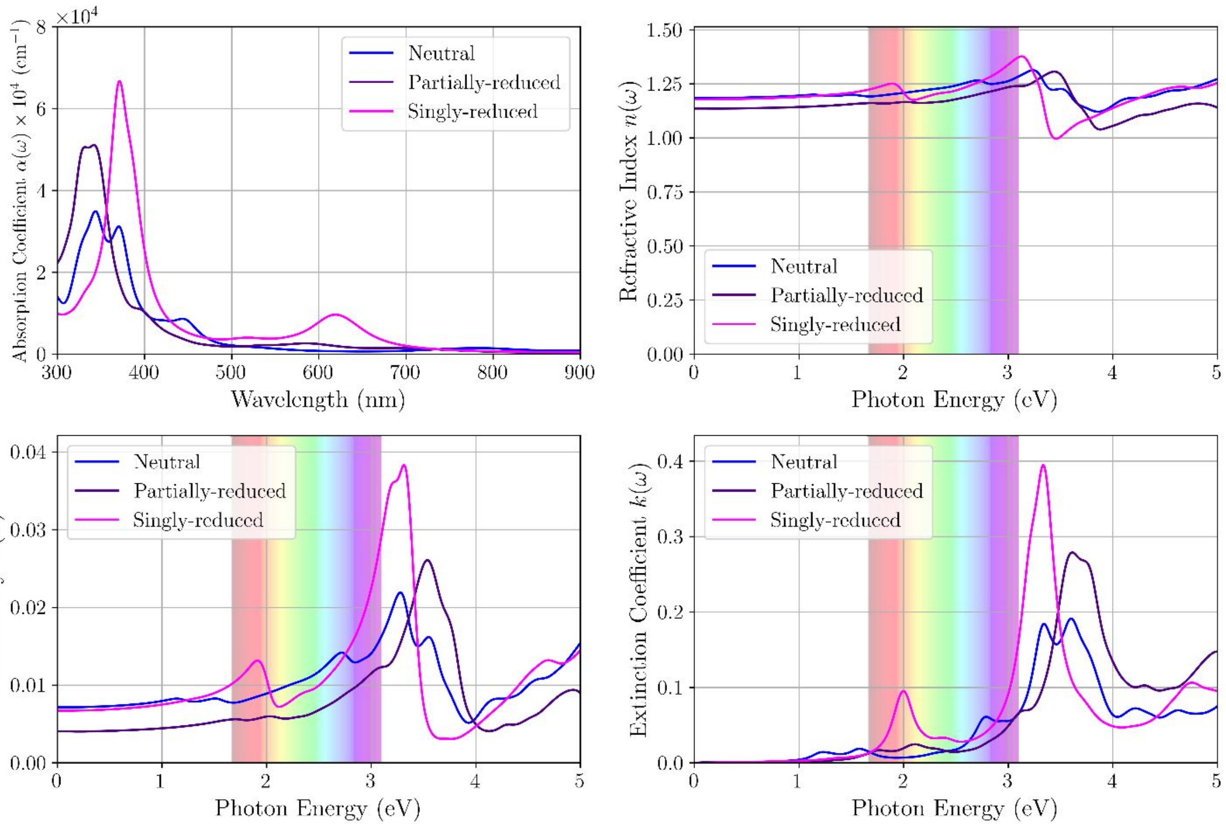

Figure S-3. *Optical properties of Zn(pyrazole-NDI) in neutral, partially-reduced, and singly-reduced states, obtained from the final frame of a 5000 fs AIMD simulation. (Top left) Absorption coefficient α(ω) as a function of wavelength, showing π–π* transitions in the neutral state and emerging absorption features upon reduction. (Top right) Refractive index n(ω) as a function of photon energy, highlighting modifications in electronic polarization induced by charge accumulation. (Bottom left) Reflectivity R(ω), demonstrating increased reflectance in the reduced states. (Bottom right) Extinction coefficient k(ω), indicating changes in optical losses across different redox states. The shaded region corresponds to the visible spectrum.*

Table S1. *Average AIM charge variations (Δq, e) per element and per functional group during the first (neutral → partially-reduced) and second (partially-reduced → singly-reduced) reductions of Zn(pyrazole–NDI).*

**Category**	*Neutral → Partially-reduced*	*Partially-reduced → singly-reduced*
**By element**		
C (48) [(a)]	+0.0047 ± 0.0848	–0.0249 ± 0.1485
H (32)	–0.0187 ± 0.0352	–0.0049 ± 0.0419
N (12)	–0.0402 ± 0.0759	+0.0475 ± 0.2449
O (8)	–0.0152 ± 0.0410	–0.0263 ± 0.0485
Zn (2)	–0.0130	–0.0052
**By functional group**		
Aromatic C (18)	–0.0097 ± 0.0707	–0.0074 ± 0.0567
Carbonyl C (8)	–0.0559 ± 0.0739	–0.0432 ± 0.3367
Carbonyl O (8)	–0.0214 ± 0.0447	–0.0308 ± 0.0447

Carbonyl group (16)	–0.0386 ± 0.0617	–0.0370 ± 0.2321
Imide N (4)	–0.0577 ± 0.0648	+0.1091 ± 0.4522
Pyrazole N (8)	–0.0314 ± 0.0836	+0.0167 ± 0.0584

[a] *Numbers in parentheses indicate the total number of atoms of each type considered in the averaging.*

Extended Analysis of Redox-Induced Structural Reorganization (Figure 4)

The evolution of three key structural parameters in the Zn(pyrazole-NDI) MOF was analyzed during AIMD simulations for the three redox states (neutral, partially-reduced, and singly-reduced) to understand the effects of electronic reduction on the material's structure (Figure 4). These parameters include: a) the dihedral angle between the planes of the pyrazole-NDI linkers and the Zn metal nodes along the y-axis (Figure 4a), b) the intermolecular O-O distance between NDI linkers along the x-axis (Figure 4b), and c) the angle between two neighboring pyrazole-NDI linkers connected to the same Zn metal node (Figure 4c), oriented along the y- and z-axes, respectively.

The ∠O-O-Zn-Zn dihedral angle, displayed in Fig. 3a, was chosen because the O atoms in the carbonyl groups are coplanar with the aromatic system of the ligand, making it a key structural descriptor. In the neutral state (purple curve), the angle fluctuates between a maximum of 77.34° (at 1624 fs) and a minimum of 32.63° (at 4631 fs). These moderate oscillations indicate a stable structure with balanced interactions between the ligands and the metal nodes. In the partially reduced state (pink curve), the ∠O–O–Zn–Zn dihedral angle spans a wide dynamic range, from a maximum of 65.77° (at 4090 fs) down to a global minimum of 22.90° (at 4828 fs). Throughout most of the trajectory, the angle exhibits broad fluctuations characteristic of the enhanced structural flexibility induced by one-electron reduction. Within the interval 1.10–3.20 ps, the system displays a region of comparatively reduced angular variability, where the dihedral oscillates predominantly between 45–55°, rather than sampling the full amplitude observed elsewhere. This interval does not constitute a true plateau, but it reflects a transiently stabilized coordination environment following the first reduction, in which the redistributed electronic density partially constrains thermally driven distortions. Outside this region, the angle explores much larger amplitudes, culminating in the global minimum of ~22.9° at 4.82–4.84 ps, which marks the strongest tendency toward coplanarity observed in the partially reduced state.

The redistribution of electron density onto the carbonyl O atoms ligands in the partially reduced state also modulates local non-covalent contacts that, while not dictating the global structural response, contribute to the framework's adaptability. When the ∠O–O–Zn–Zn dihedral angle approaches its lower values (typically in the ≤45° range), the carbonyl O atoms of one NDI linker transiently come within ≈2.6 Å of the CH_3 hydrogen atoms on a neighboring pyrazole unit along the x direction (distances consistent with weak hydrogen-bond-like O···H interactions). These short-range contacts tend to appear in conjunction with reduced dihedral angles and arise from the localized accumulation of electron density upon one-electron reduction, which enhances electrostatic attraction and transiently stabilizes low-angle geometries. Although adjacent linkers in the MOF cannot engage in direct π–π overlap due to their spatial separation, such non-covalent O···H interactions provide a secondary, charge-sensitive mechanism for structural alignment and electronic stabilization, complementing the larger-scale dihedral/O···O rearrangements that characterize the partially reduced state.

In the singly-reduced state (Fig. 4a green curve), the dihedral angle oscillates between 67.76° (at 173 fs) and 42.23° (at 833 fs), with a narrower range compared to the partially-reduced state. This indicates efficient structural and electronic reorganization to accommodate the second e^-, maintaining overall MOF stability. Despite the charge accumulation on carbonyl O atoms, which would be expected to result in smaller angles, the average dihedral angle is 52.4°, with an average O–H distance of 3.1 Å between carbonyl O atoms and CH_3 H atoms on adjacent pyrazole linkers along the x direction. This behavior arises from a contraction of the N=N bond in the pyrazole ring, decreasing from 1.43 Å to 1.38 Å. This contraction induces a geometric distortion that propagates to nearby C–C bonds and displaces the CH_3 group out of the pyrazole plane, coupling electronic redistribution with structural reorganization. As a result, the O–H distance increases, weakening the H-bond interaction. Additionally, the N-Zn bond elongates from 2.08 Å to 2.17 Å upon addition of the second e^-, pushing adjacent linkers farther apart due to the rigid tetrahedral coordination of Zn. This displacement reduces inter-linker proximity and destabilizes the corresponding H-bonds, contributing to larger dihedral angles and weaker inter-linker interactions.

Figure 4b illustrates the evolution of the intermolecular O-O distance between NDI ligands aligned in the z-direction during AIMD simulations across the three redox states. From this point forward, the xz-plane is referred to as the "nodal plane" because the Zn metal nodes are contained within this plane. It is worth noting that the ligands analyzed in this metric do not coincide with those involved in the dihedral angle analysis previously discussed (Figure 4a). In the neutral state (purple curve), the O-O distance oscillates between 4.71 Å (e.g., at ca. 2100 fs) and 8.95 Å (e.g., at ca. 4000 fs). These fluctuations reflect a flexible structure where coplanar configurations of the NDI ligands in the nodal plane are not favored. This behavior could be attributed to the lack of additional electronic interactions that stabilize the ligands proximity in this redox state.

In the Zn(pyrazole-NDI$\bullet^{-0.5}$) state, the O···O distance (pink curve, Figure 4b) displays a markedly broader oscillatory range, spanning from 4.73 Å up to 10.22 Å. The largest separations consistently occur within the same time windows in which the ∠O–O–Zn–Zn dihedral angle reaches its lowest values (Figure 4a), reflecting the moderate but systematic correlation quantified by a Pearson coefficient of $r = 0.447$. This coefficient was computed directly from the time series of ∠O–O–Zn–Zn and O···O values using the standard NumPy implementation. These coupled fluctuations indicate that transient increases in coplanarity of the NDI linker aligned along the y direction (small dihedral angles) tend to be accompanied by an expansion of the intermolecular O···O separation of the orthogonal NDI linker oriented along z. This behavior mirrors the cooperative structural events identified at ~1.10–1.18 ps, 1.33–1.37 ps, 2.8–3.0 ps, 4.3–4.4 ps and 4.82–4.84 ps, where dihedral-angle minima coincide with some of the highest O···O maxima in the trajectory.
Such coordination of motions suggests an electronically mediated structural coupling between linkers attached to the same Zn node but oriented along orthogonal crystallographic directions. Rather than merely alleviating steric contacts between carbonyl O atoms and neighboring pyrazole groups, the reorientation optimizes the distribution of excess electron density introduced upon reduction. Geometrically, larger O···O separations correspond to configurations in which the π-system of the z-oriented NDI linker becomes more closely aligned with the xz nodal plane, while the y-oriented linker approaches coplanarity with the xy plane.

In the singly-reduced state (green curve, Fig. 4b), the O-O distance oscillates between 4.86 Å (at 33 fs) and 9.51 Å (at 4632 fs). Although a trend toward larger separations is observed, this increase is smaller compared to the partially-reduced state and can be explained by the additional charge accumulation on the ligands. Unlike the Zn(pyrazole-NDI$\bullet^{-0.5}$) state, where structural reorganization

facilitates electronic coupling, in the Zn(pyrazole-NDI•⁻) state, no clear correlation is detected between the O-O distance maxima and the ∠O-O-Zn-Zn angle (green curve, Figure 4a), suggesting weaker electronic interactions between ligands. At the maximum O-O separation (4632 fs), the associated dihedral angle is 51.9°, confirming that, in this state, the ligands are oriented in planes inclined relative to the nodal plane. This structural arrangement appears to minimize electronic strain between redox centers, particularly in the vertex regions where pyrazole linkers connect.

Across all three reduction states, the π-system of NDI and the pyrazole groups remain in distinct planes within each ligand, alleviating electronic strain between NDI carbonyls and pyrazole methyl groups. In the singly reduced state, in particular, the O–O separation between NDI linkers along the z-axis (green curve, Fig. 4b) appears to be limited by non-covalent interactions detected between carbonyl O atoms and CH_3 hydrogen atoms in adjacent pyrazole linkers along the x direction, as evinced by an O-H distance of 3.77 Å at 4632 fs (along with a displacement of the O atom out of the π-plane). Additionally, an O-O repulsion is observed between carbonyl groups of adjacent ligands connected to the same Zn node but oriented in orthogonal directions (z and y). A carbonyl group that gains e^- density upon double reduction may become more susceptible to repulsion from neighboring carbonyls. This effect, combined with the rigidity of the tetrahedral Zn environment, could prevent optimal alignment between the π-systems of adjacent linkers in different directions ($\varphi_{(\pi_1-\pi_2)} = 0$ and small dihedral angle ∠O–O–Zn–Zn). As a result, lower conductivity is observed in this singly reduced state. In fact, the maximum O-O distance between adjacent linkers in different directions (different than the O–O distance plotted in Fig. 4b) is 4.64 Å and 4.38 Å at the points of greatest separation within the simulation for the Zn(pyrazole-NDI•$^{-0.5}$) and Zn(pyrazole-NDI•⁻) states, respectively, indicating that the O-H non-covalent interaction is stronger than the repulsion between reduced carbonyls.

Computational Details for Electron Transfer Coupling (Figure 5a)

The *Electron Transfer Coupling Energy* was evaluated as a function of the dihedral angle $\varphi(\pi_1-\pi_2)$ in the Zn(pyrazole–NDI) MOF, as reported in Figure 5a. The calculations were performed on a finite atomic cluster, where hydrogen atoms were added to complete the connectivity and emulate the behavior of two connected linkers within a periodic environment of the crystal lattice. Partial geometry optimizations were carried out in Gaussian 16 for selected values of $\varphi_{(\pi_1-\pi_2)}$, ensuring structural consistency. Subsequently, constrained Density Functional Theory (cDFT)[21] calculations were performed using the NWChem software[22] to evaluate the Electron Transfer Coupling Energy for each optimized configuration.

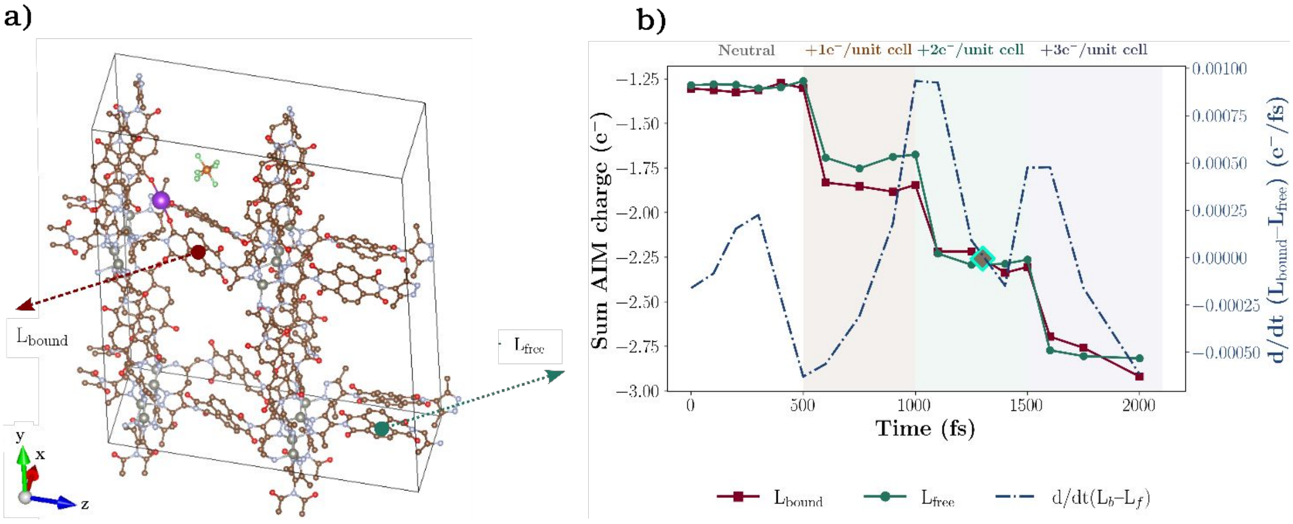

Figure S-4. *Correlation between the structural environment of K⁺ and charge localization in Zn(pyrazole–NDI) MOF. (a) Supercell snapshot highlighting two representative linkers: one directly coordinated to K⁺ and*

involved in trimer formation ($L_{bound,}$ red), and a distant, unbound linker (L_{free}, green). (Right) Time evolution of the summed AIM charges for L_{bound} (red) and Lfree (green) during successive electron additions under AIMD at 300 K. In the +1e⁻/unit cell regime (x ≈ 0.5), L_{bound} accumulates more electronic density than L $_{free}$, revealing symmetry breaking consistent with mixed-valence behavior. This asymmetry vanishes in the +2e⁻ and +3e⁻/unit cell regimes, where electron distribution becomes more uniform. The dashed blue curve tracks the time derivative of the charge imbalance ($L_{bound,}$ $-_{free}$), serving as a proxy for the temporal sensitivity of valence symmetry breaking. Notably, the imbalance is most pronounced in the Zn(pyrazole–NDI•⁻0.5) regime, underscoring its mixed-valence character.

Hybrid functional validation

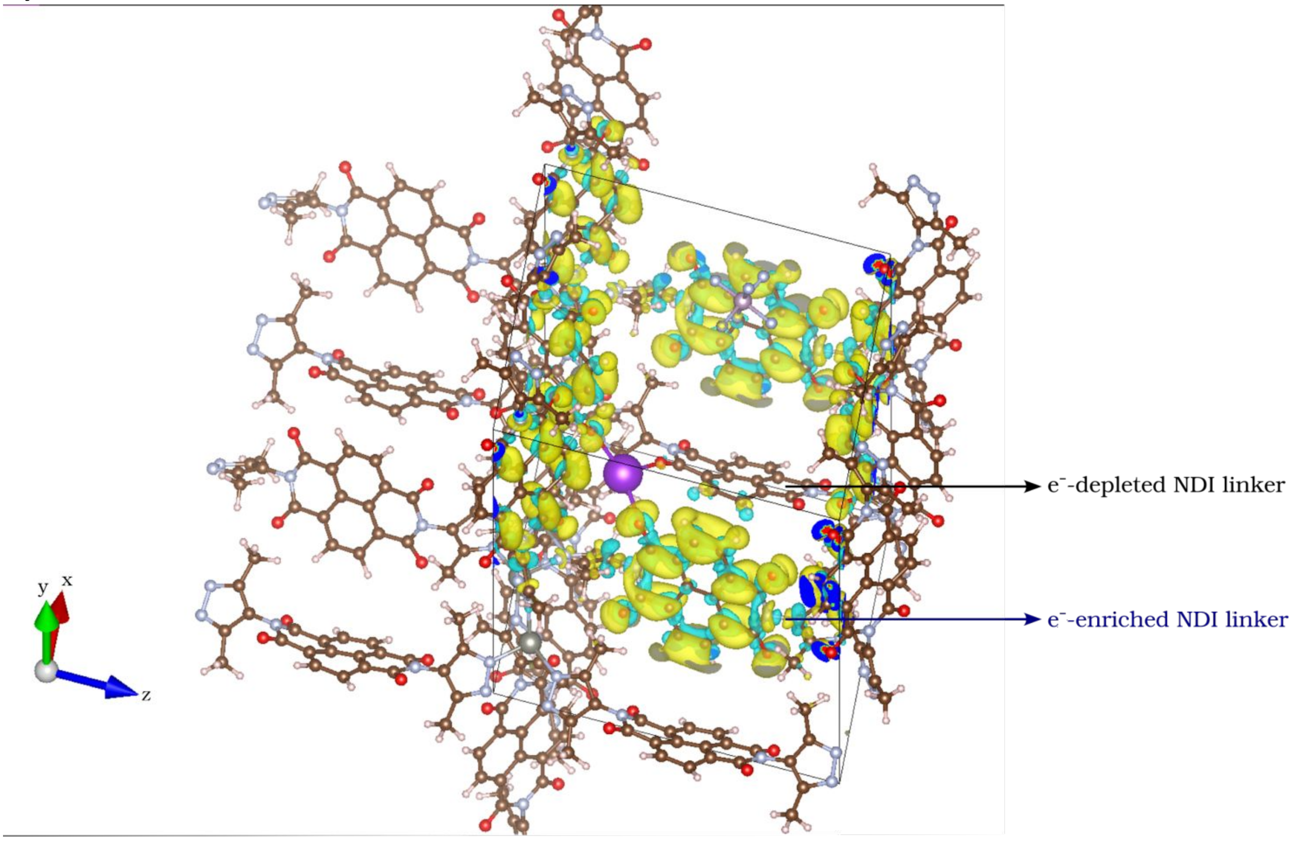

Figure S-5. *Hybrid-DFT validation of symmetry-broken charge localization in the partially reduced Zn(pyrazole-NDI•-0.5) framework. Charge-density-difference isosurface, $\Delta\rho=\rho_{reduced}-\rho_{neutral}$ obtained from single-point HSE06 (25% exact exchange) calculations performed on the snapshot corresponding to the maximum-stability trimer configuration in the Zn(pyrazole-NDI•-0.5) state, using the same periodic geometry in the presence of K^+. The map reveals an asymmetric distribution of the excess electron density between the two z-oriented NDI linkers: one linker shows substantially greater charge accumulation (electron-enriched NDI linker), whereas the other exhibits only a much weaker density increase (electron-depleted NDI linker). This hybrid-functional result is consistent with a mixed-valence, symmetry-broken charge distribution stabilized by the local K^+ coordination environment. Isosurface value = 0.014 $e^-/Å^3$.*

We performed a single-point hybrid-DFT validation at the HSE06 level (25% exact exchange) on the same fixed periodic geometry used in the AIMD/PMF analysis, namely the 2×1×1 minimal supercell containing one $K[PF_6]$ unit, and corresponding to the maximum-stability trimer snapshot in the Zn(pyrazole-NDI•-0.5) regime (Figure S-5). This is the most direct test of whether the reported mixed-valence state is a semilocal-functional artifact. The result shows that the same qualitative symmetry-broken charge distribution is preserved at the hybrid level. One z-oriented NDI linker remains significantly more electron-enriched,

whereas the other remains relatively electron-depleted, consistent with the PBE description. Thus, the central conclusion is not removed by inclusion of exact exchange: the mixed-valence pattern is qualitatively robust and is stabilized by the local K^+ coordination environment. Importantly, we also find that at the same HSE06 level (25% exact exchange) the excess e^- remains delocalized within the unit cell of the corresponding K^+-free framework (as shown for the x = 0.5 state in Fig. 3 of the main manuscript). This point is critical because it shows that the symmetry-broken charge-localization pattern is preserved beyond the specific functional treatment, and it is associated with formation of the K^+-mediated trimer environment, which stabilizes the asymmetric mixed-valence charge distribution.
This result is directly relevant to the prediction of K^+-mediated trimer formation, because the validated hybrid snapshot is precisely the maximum-stability trimer configuration identified in the partially reduced regime. In other words, the hybrid benchmark does not merely confirm linker-centered reduction in general; it confirms the electronically asymmetric, K^+-coupled trimer environment that underlies the mechanistic interpretation of the manuscript.
Regarding the basis set, these periodic calculations were performed in VASP within a plane-wave PAW framework, so the relevant numerical controls are the plane-wave cutoff and the PAW description, rather than an atom-centered basis set in the molecular sense. Once numerically converged, such basis effects are not expected to change the qualitative identification of the mixed-valence linker manifold or the K^+-bridged trimer motif.
Regarding simulation temperature, the trimer motif was not inferred from a single optimized structure, but from finite-temperature AIMD/PMF sampling. Temperature will certainly affect relative populations, lifetimes, and free-energy differences among coordination states. However, the underlying physical tendency, K^+ bridging adjacent reduced NDI environments and stabilizing a symmetry-broken mixed-valence state, emerges from the trajectory analysis itself and is therefore not a fragile consequence of a single temperature point.

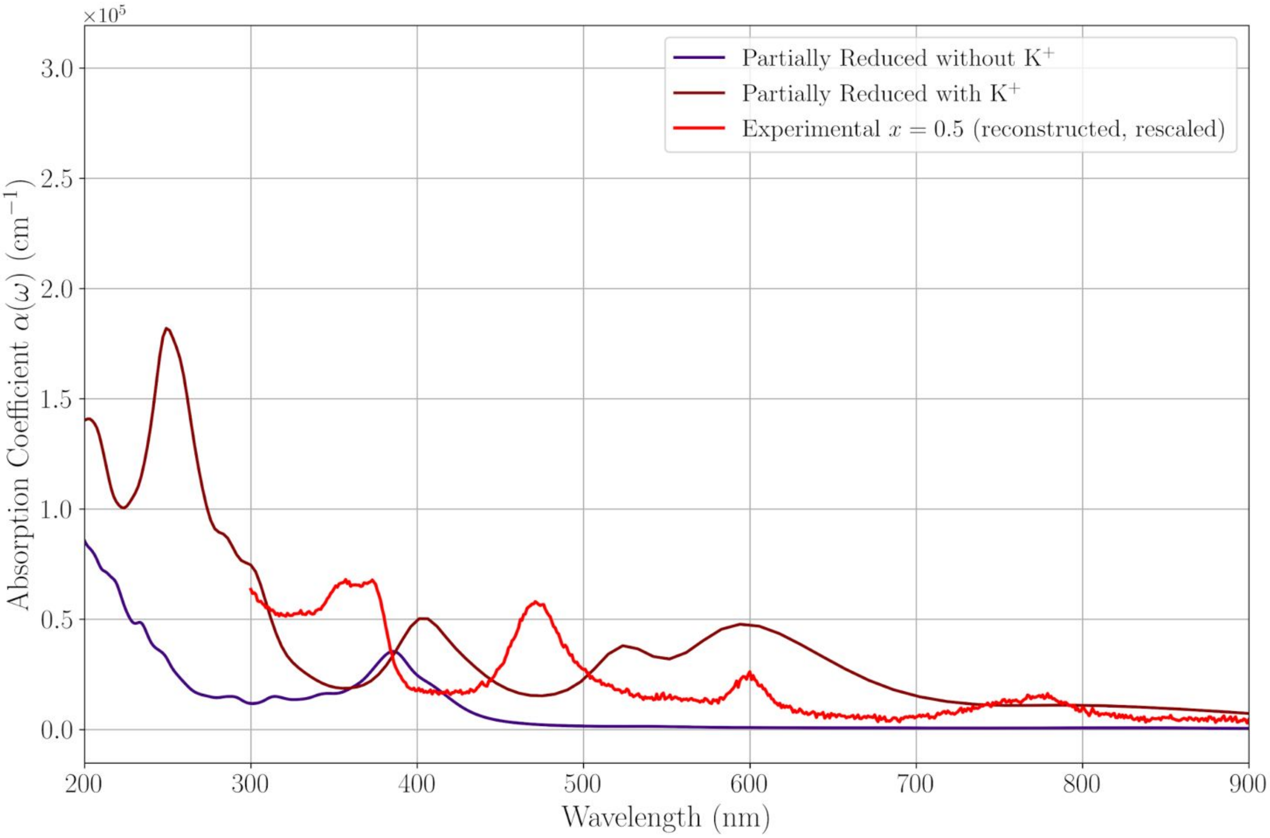

Figure S-6. *Calculated UV–vis absorption spectra for the partially reduced Zn(pyrazole-NDI) framework obtained without K^+ (indigo curve) and with explicit K^+ (dark red curve, K^+-bridged trimer, fixed AIMD*

snapshot at 2595 fs), compared with the published experimental Spectro electrochemical profile for the half-reduced state (x=0.5) shown as the red curve, reconstructed from the literature[5] and rescaled for qualitative comparison of spectral shape. The model including explicit K^+ reproduces the experimental lineshape more closely, particularly the absorptions above 400 nm, which are not recovered in the calculation without K^+. The three main features of the K^+-containing model, located at 406.48, 523.58, and 594.07 nm, are assigned to a NDI π–π∗ excitation and two NDI·--derived excitations related to the experimental bands, respectively. Experimental data adapted and reconstructed from [5]. Available under a CC-BY [4.0] International License. Copyright [2023] [Li, J.; Kumar, A.; Johnson, B. A.; Ott, S.]

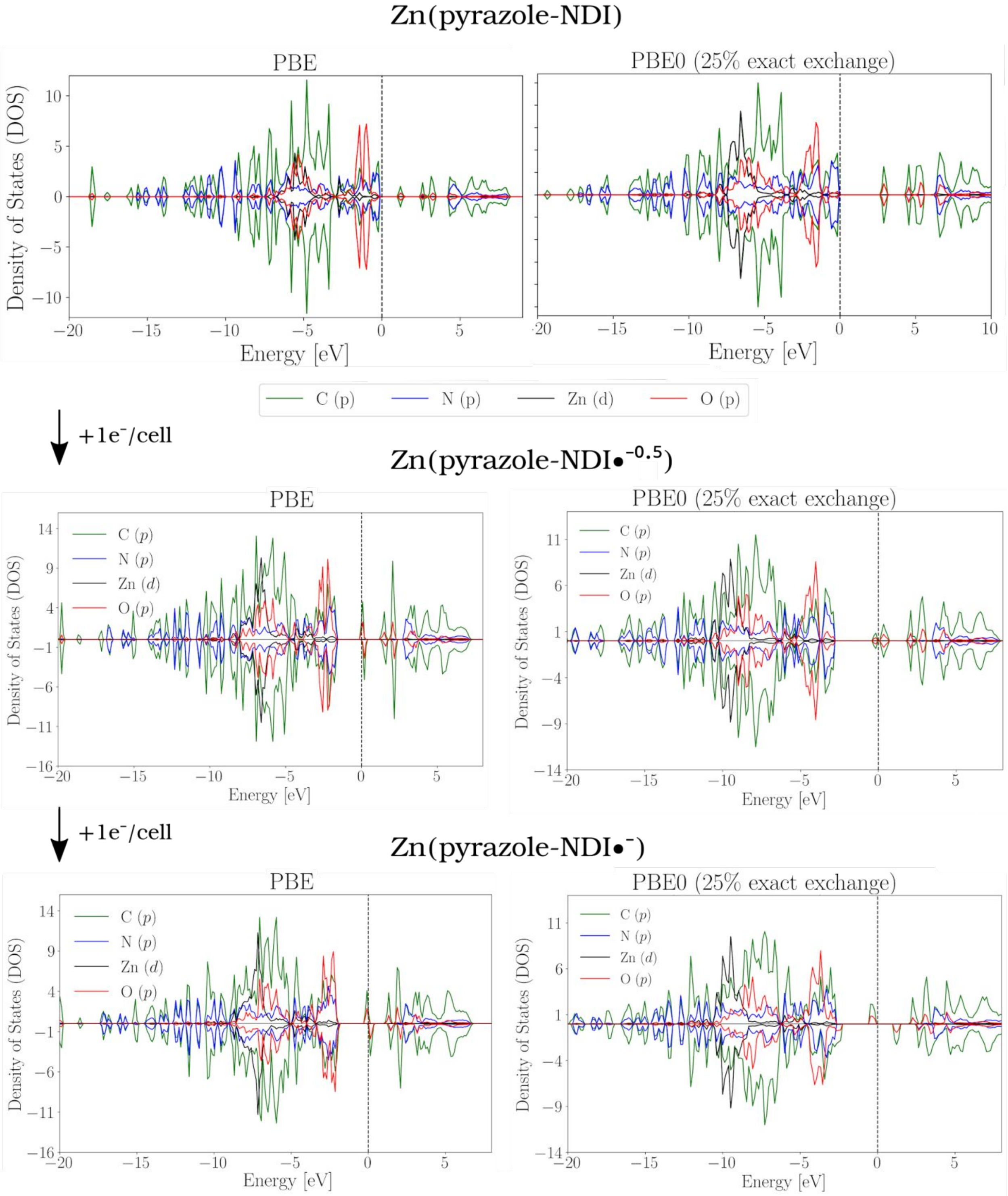

Figure S-7. *Projected density of states (PDOS) of Zn(pyrazole-NDI) in the neutral, partially reduced, and singly reduced unit-cell configurations. The left and right columns correspond to PBE and PBE0 (25% exact*

exchange), respectively, evaluated on the same fixed geometry for each redox state. Shown are the C p, N p, O p, and Zn d contributions. Energies are referenced to the Fermi level (Ef =0 eV), such that negative and positive energies correspond to states below and above Ef , respectively. Positive and negative DOS values denote the spin-up and spin-down channels, respectively. Relative to PBE, PBE0 increases the occupied–unoccupied separation while preserving the qualitative frontier-state character. Across all three redox states, the states relevant to reduction remain predominantly ligand-centered, with dominant C/N/O contributions, whereas Zn d states do not emerge as the primary low-energy acceptor manifold.

Ion Concentration and Trimer-Mediated Charge Dynamics

In principle, each injected electron should be balanced by a counterion to preserve overall charge neutrality. However, in our AIMD simulations the introduction of a single K[PF_6] unit into the neutral 2×2×2 supercell of Zn(pyrazole–NDI) (unit-cell volume ≈ 14,939 Å^3; supercell volume ≈ 119,514 Å^3, equivalent to 1.20×10^{-22} L) corresponds to an effective concentration of ~0.014 M K^+. By contrast, strict charge compensation for +8, +16, and +24 e^- injections would require 8, 16, and 24 K^+ ions, corresponding to ~0.11, 0.22, and 0.33 M, respectively.

We deliberately maintained only one explicit counterion throughout the reduction sequence. This strategy was chosen to (i) impose a locally elevated counterion concentration while remaining within the same order of magnitude as experimentally relevant regimes, and (ii) isolate the structural and electronic role of K^+ coordination without conflating the results with additional complexity arising from changing counterion stoichiometries. Accordingly, this approach should be regarded as a simplified but informative model, aimed at probing the localized influence of K^+ under stepwise electronic reduction rather than reproducing exact experimental charge neutrality.

AIMD-Optimized Unit Cell Structure

The atomic coordinates of the Zn(pyrazole-NDI) MOF unit cell, corresponding to the final configuration after 5000 fs of AIMD simulation at 300 K, are provided below in POSCAR format to facilitate reproducibility.

POSCAR file written by OVITO Basic 3.12.2
1
7.520337 0.898748 -0.767316
1.411398 17.200021 -0.084662
-1.295252 -0.156256 17.16224
C N Zn O H
48 12 2 8 32
Cartesian
3.5443291295 1.5785552846 15.8155267529
5.5378225598 2.5586041897 15.9239467834
4.6671777739 1.9638755704 15.0628347804
5.8353642166 1.2070136704 13.0940294963
6.2813906661 1.5438609424 11.7502452743
5.5702171683 2.5454171417 11.0301581841
4.6273396058 3.353843649 11.6393560431
4.252646836 3.2268224826 13.0605639414
5.7093326986 2.6104839097 9.6353933239
4.7972315595 3.4528443429 8.8840789331
3.7839931843 4.1418620063 9.4861469777
3.7470928353 4.1160568979 10.8994469335

6.5348422809 1.7192635323 8.9372342838
6.7413085516 1.7474944984 7.4727758089
4.9240725013 3.4306381991 7.4111501232
1.0840149441 17.0558775532 11.7652688159
1.1715730005 17.1652026326 10.3566110137
5.6717618519 2.5497931675 5.3546370362
4.712253902 1.9695497899 4.4792884146
6.6095993613 3.20226163 4.5008788096
0.8914840471 4.9532119977 2.3275264079
1.9647941 5.7134770832 1.8698166455
2.9668526098 4.871497984 1.3659930967
4.3549162173 5.2079232626 1.0100887441
3.0804395651 7.6245982978 2.6669502864
3.1421944951 9.0583267566 2.8360129492
2.4704262844 9.8705229323 1.8494838906
1.4927716369 9.3528111828 0.9173756952
1.1990882451 7.8691873837 0.9575113459
2.7596548749 11.285581743 1.7484661852
3.7285980944 11.8129086989 2.6204839379
4.3159458728 11.0050441632 3.6272246038
4.0351642964 9.6538947217 3.7607757615
1.9801778976 12.1341791401 0.8524548672
8.4927242685 12.4810230352 -0.7350798783
8.2785495518 11.1013522357 -0.6998046773
2.1470281186 13.6531392354 1.0022919805
4.0997826121 13.2586631512 2.5890126006
3.3486196409 15.5186536915 1.9644088441
2.3983228343 16.3638164673 2.532709438
4.321889781 16.3826263168 1.3732512032
7.0707360166 6.3484950443 2.1509143795
1.1347343399 16.0425861303 3.3178542451
5.4860671411 16.0455739745 0.549062548
3.5550901238 1.0919851438 4.8326528119
0.1906320297 3.1864385497 5.7822722307
-0.7768963911 2.5111411161 16.3396808349
2.3432793934 0.9009721804 15.271826926
5.0978817143 2.0187737011 -0.0432115538
6.3532060388 2.6273306674 0.0428481738
4.9717843798 2.1315031369 13.6687234292
5.6668422807 2.4386971183 6.7900330316
5.1141396153 2.1586550425 3.1823888717
6.2370026812 2.9560542046 3.1992325526
2.5515006194 3.6134224 1.6412138801
1.2539718624 3.6249926383 2.1466979056
2.0892751353 7.0963178745 1.7965701794
3.169657482 14.1154333737 1.9032927605
1.3892300824 0.4517582493 2.4678528148
2.6021461699 0.4888107472 1.6826594134
3.8378770495 2.0623497529 1.6564170075
0.1009784295 1.8761222865 2.3692818747
3.8612669448 6.8469986781 3.2659249103
0.3394318973 7.2900490662 0.3388392696
1.3768319101 14.4562477532 0.4632860008
5.0689722687 13.6206047201 3.2512867385

4.383302842 4.3522333408 6.7465238001
1.5180616431 17.5131936731 7.4919189303
7.5626246968 17.3681861108 13.5381092457
3.3554482327 3.7958714626 13.6591372134
6.4723722604 6.8710334744 1.4000090398
6.5426749251 5.5519453946 2.6818797541
7.3402993767 7.1375066217 2.8747841572
4.3943964586 6.0778360588 0.3389806581
3.5054559311 5.2721773735 19.1458038455
4.8605912199 4.4319179059 0.432286194
0.5708054271 15.3077720498 2.7246737969
8.9460365009 16.4560429406 3.4941070249
8.0149048615 17.8083106901 2.7445226229
3.9345965868 16.1460981002 16.6750025223
6.3635231603 16.6116115975 0.9120942959
5.7676126251 14.9836032354 0.553903468
6.2236673074 10.4602428654 15.8671476116
4.5350777894 9.1149685431 4.5544913016
5.0168702611 11.4556398116 4.3280251129
6.6600269196 12.9793529037 15.7268986679
3.6419984445 0.0373020833 4.544436873
3.4553230784 1.0864209478 5.9357105685
3.9795002347 18.6488238986 4.4146482943
2.9828979695 4.6000340915 8.8787591189
1.7278637583 16.415698825 9.8501967402
2.9438477794 4.5811258663 11.4475940259
1.6317809839 16.235073788 12.2591370792
0.1458005167 2.321312497 16.9081268693
7.056378451 3.2832088069 14.5286026374
0.3143168178 3.7171694129 -0.5537244618
1.5646701388 0.8262421099 16.0363956027
2.0257899803 1.4793847081 14.3998059124
2.6026503502 -0.1357218746 15.029819585
0.730044662 3.712350816 4.977229855
1.0218453581 2.6611119803 6.2875636382
-0.2622438492 3.9339317391 6.47184666

References

(1) Hafner, J. Ab-initio simulations of materials using VASP: Density-functional theory and beyond. *Journal of computational chemistry* **2008**, *29* (13), 2044-2078.
(2) Paier, J.; Hirschl, R.; Marsman, M.; Kresse, G. The Perdew–Burke–Ernzerhof exchange-correlation functional applied to the G2-1 test set using a plane-wave basis set. *The Journal of chemical physics* **2005**, *122* (23).
(3) Sun, G.; Kürti, J.; Rajczy, P.; Kertesz, M.; Hafner, J.; Kresse, G. Performance of the Vienna ab initio simulation package (VASP) in chemical applications. *Journal of Molecular Structure: THEOCHEM* **2003**, *624* (1-3), 37-45.
(4) Martyna, G. J.; Klein, M. L.; Tuckerman, M. Nosé–Hoover chains: The canonical ensemble via continuous dynamics. *The Journal of chemical physics* **1992**, *97* (4), 2635-2643.

(5) Li, J.; Kumar, A.; Johnson, B. A.; Ott, S. Experimental manifestation of redox-conductivity in metal-organic frameworks and its implication for semiconductor/insulator switching. *Nature Communications* **2023**, *14* (1), 4388.
(6) Tang, W.; Sanville, E.; Henkelman, G. A grid-based Bader analysis algorithm without lattice bias. *Journal of Physics: Condensed Matter* **2009**, *21* (8), 084204.
(7) Sanville, E.; Kenny, S. D.; Smith, R.; Henkelman, G. Improved grid-based algorithm for Bader charge allocation. *Journal of computational chemistry* **2007**, *28* (5), 899-908.
(8) Henkelman, G.; Arnaldsson, A.; Jónsson, H. A fast and robust algorithm for Bader decomposition of charge density. *Computational Materials Science* **2006**, *36* (3), 354-360.
(9) Stukowski, A. Visualization and analysis of atomistic simulation data with OVITO–the Open Visualization Tool. *Modelling and simulation in materials science and engineering* **2009**, *18* (1), 015012.
(10) Roux, B. The calculation of the potential of mean force using computer simulations. *Comput. Phys. Commun.* **1995**, *91* (1), 275-282. DOI: https://doi.org/10.1016/0010-4655(95)00053-I.
(11) Schneider, T.; Stoll, E. Molecular-dynamics study of a three-dimensional one-component model for distortive phase transitions. *Physical Review B* **1978**, *17* (3), 1302.
(12) Wu, Q.; Van Voorhis, T. Constrained Density Functional Theory and Its Application in Long-Range Electron Transfer. *J. Chem. Theory Comput.* **2006**, *2* (3), 765-774. DOI: 10.1021/ct0503163.
(13) Wu, Q.; Van Voorhis, T. Direct optimization method to study constrained systems within density-functional theory. *Physical Review A* **2005**, *72* (2), 024502. DOI: 10.1103/PhysRevA.72.024502.
(14) Valiev, M.; Bylaska, E. J.; Govind, N.; Kowalski, K.; Straatsma, T. P.; Van Dam, H. J. J.; Wang, D.; Nieplocha, J.; Apra, E.; Windus, T. L.; et al. NWChem: A comprehensive and scalable open-source solution for large scale molecular simulations. *Comput. Phys. Commun.* **2010**, *181* (9), 1477-1489. DOI: https://doi.org/10.1016/j.cpc.2010.04.018.
(15) Marcus, R. A. On the Theory of Oxidation-Reduction Reactions Involving Electron Transfer. I. *The Journal of Chemical Physics* **1956**, *24* (5), 966-978. DOI: 10.1063/1.1742723 (acccessed 10/31/2025).
(16) Marcus, R. A. Chemical and Electrochemical Electron-Transfer Theory. *Annu. Rev. Phys. Chem.* **1964**, *15* (Volume 15), 155-196. DOI: https://doi.org/10.1146/annurev.pc.15.100164.001103.
(17) Günther, D.; Boto, R. A.; Contreras-Garcia, J.; Piquemal, J. P.; Tierny, J. Characterizing Molecular Interactions in Chemical Systems. *IEEE Transactions on Visualization and Computer Graphics* **2014**, *20* (12), 2476-2485. DOI: 10.1109/TVCG.2014.2346403.
(18) Lu, T.; Chen, F. Multiwfn: A multifunctional wavefunction analyzer. *J. Comput. Chem.* **2012**, *33* (5), 580-592. DOI: https://doi.org/10.1002/jcc.22885.
(19) Pettersen, E. F.; Goddard, T. D.; Huang, C. C.; Couch, G. S.; Greenblatt, D. M.; Meng, E. C.; Ferrin, T. E. UCSF Chimera—A visualization system for exploratory research and analysis. *J. Comput. Chem.* **2004**, *25* (13), 1605-1612. DOI: https://doi.org/10.1002/jcc.20084.
(20) Momma, K.; Izumi, F. VESTA: a three-dimensional visualization system for electronic and structural analysis. *Applied Crystallography* **2008**, *41* (3), 653-658.
(21) O'Regan, D. D.; Teobaldi, G. Optimization of constrained density functional theory. *Physical Review B* **2016**, *94* (3), 035159.
(22) Valiev, M.; Bylaska, E. J.; Govind, N.; Kowalski, K.; Straatsma, T. P.; Van Dam, H. J. J.; Wang, D.; Nieplocha, J.; Aprà, E.; Windus, T. L. NWChem: A comprehensive and scalable open-source solution for large scale molecular simulations. *Computer Physics Communications* **2010**, *181* (9), 1477-1489.
